# TMEM209 promotes hepatocellular carcinoma progression by activating the Wnt/β-catenin signaling pathway through KPNB1 stabilization

**DOI:** 10.1038/s41420-024-02207-9

**Published:** 2024-10-16

**Authors:** Haoran Fang, Xiaoyi Shi, Jie Gao, Zhiping Yan, Yun Wang, Yabin Chen, Jiacheng Zhang, Wenzhi Guo

**Affiliations:** 1 Department of Hepatobiliary and Pancreatic Surgery, The First Affiliated Hospital of Zhengzhou University, Zhengzhou, China; 2 Henan Engineering & Research Center for Diagnosis and Treatment of Hepatobiliary and Pancreatic Surgical Diseases, Zhengzhou, China; 3 Henan Key Laboratory of Digestive Organ Transplantation, Zhengzhou, Henan China

**Keywords:** Oncogenes, Metastasis

## Abstract

Hepatocellular carcinoma (HCC) is the most common malignancy in the liver, with a poor prognosis. Transmembrane protein 209 (TMEM209) involves multiple biological processes, such as substance transportation and signal transduction, and is abundantly expressed in tumor tissues. However, the relationship between TMEM209 and HCC has not been comprehensively elucidated. In this study, we aimed to illustrate this issue by in vitro *and* in vivo experiments. Bioinformatic analysis and clinical sample validation revealed that TMEM209 was upregulated in HCC and correlated with reduced survival duration. Functionally, TMEM209 promoted the proliferation, migration, invasion, and EMT of HCC cells in vitro and facilitated tumor growth and metastasis in xenograft models. Mechanistically, TMEM209 promoted the proliferation and metastasis of HCC in a KPNB1-dependent manner. Specifically, TMEM209 could bind to KPNB1, thereby competitively blocking the interaction between KPNB1 and the E3 ubiquitin ligase RING finger and CHY zinc finger domain-containing protein 1 (RCHY1) and preventing K48-associated ubiquitination degradation of KPNB1. Ultimately, the Wnt/β-catenin signaling pathway was activated, contributing to the progression of the malignant phenotype of HCC. In conclusion, the molecular mechanism underlying the TMEM209/KPNB1/Wnt/β-catenin axis in HCC progression was elucidated. TMEM209 is a potential biomarker and therapeutic target for HCC.

## Background

Primary liver cancer (PLC) is the sixth most common malignancy and the third leading cause of cancer-related deaths worldwide [1]. Hepatocellular carcinoma (HCC) accounts for approximately 90% of PLC [1]. Despite recent advancements in treatment modalities, including surgeries, targeted therapy, radiotherapy, and combined therapy for HCC, the prognosis of these patients remains unsatisfactory [2, 3]. An abundant blood supply from the liver contributes to the highly proliferative and aggressive nature of HCC. Significant challenges to its clinical treatment are due to a scarce understanding of the mechanisms driving HCC progression. Consequently, there is a pressing need to thoroughly investigate the underlying mechanisms and identify novel biomarkers and therapeutic targets.

Transmembrane proteins (TMEMs), spanning the biological membranes, are integral in processes including signal transduction, catalysis, protein scaffolds, protein stability, and substance transportation [4, 5]. Although the biological functions of most TMEMs are unknown, accumulating evidence suggests that dysregulation of certain TMEMs in tumors plays oncogenic or tumor suppressive roles, thus affecting the proliferation, metastasis, and drug resistance of tumors [6–9]. As a member of TMEMs, transmembrane protein 209 (TMEM209) was initially identified as a nuclear membrane protein in the mouse liver, and its localization was subsequently observed in the Golgi apparatus, vesicles, and cytoplasm [10, 11]. High-throughput sequencing suggested the association of TMEM209 variants to lymphoma [12] and genome-wide analyses indicated that TMEM209 might be a target for clonal hematopoiesis [13]. Lung cancer studies posited that TMEM209 was highly expressed in tumor tissues and promoted their proliferation, implicating that TMEM209 may act as an oncogene [11]. Moreover, TMEM209 was implicated in protein stability regulation, particularly in increasing the protein expression of NUP205 [11]. However, research on TMEM209’s relationship with HCC is less understood, and the role and mechanism of TMEM209 in HCC remain to be elucidated.

Karyopherin-β1 (KPNB1) belongs to the importin family and plays a pivotal role in facilitating the transfer of critical protein molecules to the nucleus and affecting relevant signaling pathways [14, 15]. Moreover, KPNB1 is upregulated in various cancer tissues and contributes to tumor progression [16–18]. In glioblastoma research, KPNB1 knockdown inhibited β-catenin nuclear import, resulting in the inactivation of the Wnt/β-catenin signaling pathway and the arrest of proliferation [15], and targeting KPNB1 alleviated TRAIL resistance [19]. Interestingly, KPNB1 was overexpressed in HCC tissues and regarded as a biomarker for prognostic prediction [20]. Generally, protein overexpression is regulated by multiple levels involved in gene amplification, transcriptional activation, or degradation inhibition. Past studies indicated that certain importins were inhibited or activated by ubiquitin-proteosome pathway [21]. Whether this mechanism is involved in KPNB1 activation and the relationship between TMEM209 and KPNB1 in HCC still remains poorly understood.

Continuous activation of the Wnt/β-catenin signaling pathway majorly contributes to hepatocarcinogenesis and metastasis [22, 23]. β-catenin is the pivot protein in Wnt/β-catenin signaling pathway. Typically, β-catenin is phosphorylated at the T41/S33/37 sites and subsequently degraded by ubiquitin-proteosome pathway in cytoplasm [24]. By contrast, β-catenin becomes more stabilized upon Ser675 (S675) or Ser552 (S552) phosphorylation in cytoplasm [25–27]. Subsequently, β-catenin translocating from cytoplasm to the nucleus triggers a signal transduction cascade, promoting the transcription of its downstream genes, such as c-Myc, cyclin-D1, Axin2, GLUL, and CDK4, thereby results the activation of Wnt/β-catenin signaling [22, 28, 29]. Multiple proteins affect the Wnt/β-catenin signaling pathways, including importins and TMEMs [15]. However, their regulation mechanism of the Wnt/β-catenin signaling pathway remains unclear.

In this study, we aimed to analyze the role and mechanism of TMEM209 in HCC. Our experiments confirmed TMEM209 overexpression in HCC, which was a predictor of poor prognoses for HCC patients. TMEM209 could promote the progression of HCC through the TMEM209/KPNB1/Wnt/β-catenin axis. By addressing the above-mentioned research gap, our study provides valuable insights into the mechanisms underlying HCC progression and posits TMEM209 as a promising target for the prognosis and therapy of patients with HCC.

## Results

### TMEM209 is upregulated in HCC specimens and is a predictor of poor prognosis

**Fig. 1:**
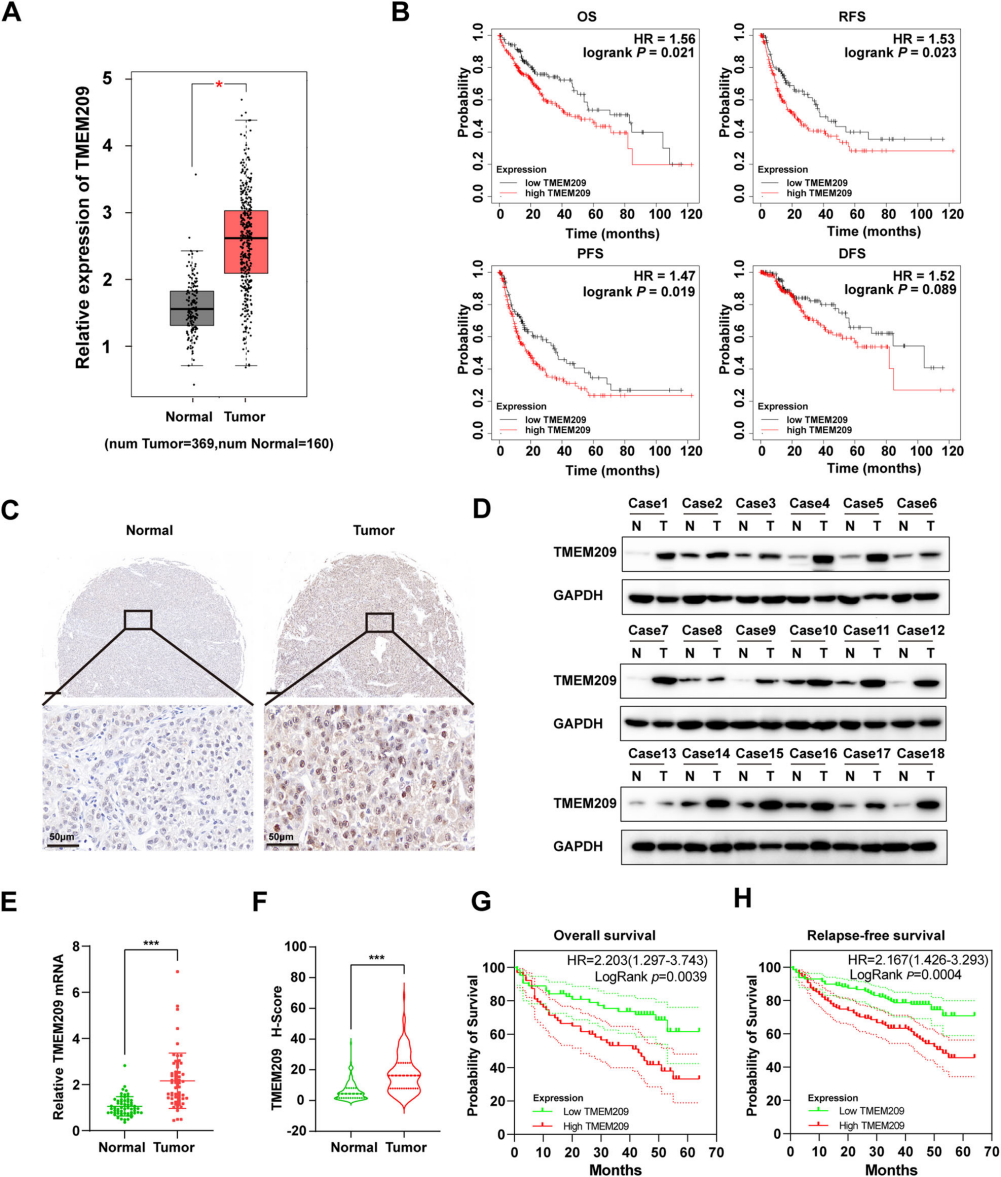
TMEM209 is upregulated in HCC and indicates poor survival. **A** The expression of TMEM209 was upregulated in HCC-TCGA and GTEx datasets. **B** High expression of TMEM209 indicated poor overall survival (OS), relapse-free survival (RFS), progression-free survival (PFS), and disease-free survival (DFS) of HCC patients in TCGA datasets. **C** Representative images of protein expression of TMEM209 in HCC tissues and adjacent normal tissues by immunohistochemistry (IHC) (Scale bar = 50 μm). **D** The protein levels of TMEM209 in HCC tissues and adjacent normal tissues (n = 18) were determined by western blotting. N = normal tissues, T = tumor tissues. **E** The mRNA levels of TMEM209 in HCC tissues and adjacent normal tissues (n = 60) were assayed by the qPCR. Data were assessed by paired-sample *t* test. **F** IHC analysis of TMEM209 expression in a microarray comprising 129 tumor tissues and adjacent normal tissues derived from patients with HCC admitted to the First Affiliated Hospital of Zhengzhou University. Data were assessed by paired-sample *t* test. Kaplan-Meier analysis of OS (**G**) and RFS (**H**) and log-rank test based on the protein expression of TMEM209 in 129 HCC patients with HCC admitted to the First Affiliated Hospital of Zhengzhou University. *P < 0.05, ***P < 0.001.

To determine the role of TMEM209 in HCC, we initially analyzed The Cancer Genome Atlas (TCGA) and genotype-tissue expression databases and found that TMEM209 was highly expressed in HCC tissues (Fig. 1A). Subsequently, survival analysis performed by the online Kaplan–Meier (KM) plotter program demonstrated a negative correlation between TMEM209 expression and the prognosis of patients with HCC, including overall survival (OS), progression-free survival (PFS), relapse-free survival (RFS), and disease-free survival (DFS) (Fig. 1B). Additionally, we collected paired HCC tissues from patients admitted at the First Affiliated Hospital of Zhengzhou University and determined the expression of TMEM209 at the protein and mRNA levels. The immunohistochemistry (IHC) analysis indicated that TMEM209 was primarily localized to the cytoplasm, nuclear envelope, and cell membrane (Fig. 1C). Western blotting and IHC revealed that TMEM209 protein expression was upregulated in HCC (Figs. 1C, D, and S1). Similarly, qPCR analysis indicated a remarkable upregulation of TMEM209 mRNA in tumor tissues (Fig. 1E). Finally, IHC staining of a tissue microarray (N = 129) showed that the relative expression of TMEM209 in tumor tissues (17.69 ± 12.72) was notably higher than that of the normal tissues (6.044 ± 5.895) (Fig. 1F). Furthermore, KM analysis based on tissue microarray indicated that patients with high TMEM209 expression exhibited relatively poor OS and RFS (Fig. 1G, H). The hazard ratio (HR) values for OS and RFS were 2.203 and 2.167, respectively. Therefore, we inferred that TMEM209 was upregulated in HCC and predicted poor prognoses for these patients.

### TMEM209 promotes the proliferation and metastasis of HCC in vitro

**Fig. 2:**
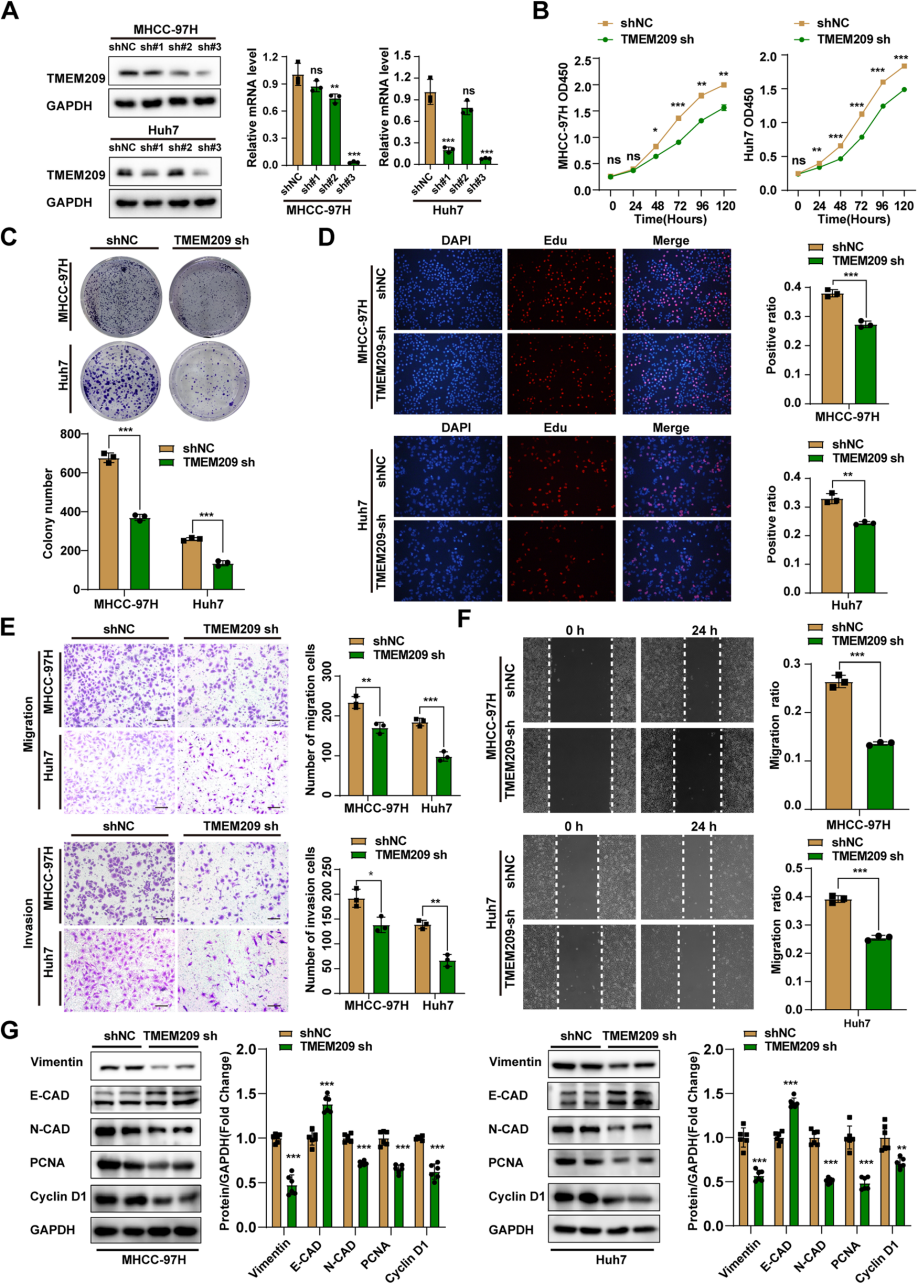
Knockdown TMEM209 inhibits the proliferation and metastasis of HCC in vitro. **A** Knockdown efficiency of TMEM209 in MHCC-97H and Huh7 cells was evaluated by western blotting and qPCR. **B** The CCK-8 assays were performed to detect the effects of TMEM209 knockdown on the proliferation efficiency of MHCC-97H and Huh7 cells. **C** Representative images (upper) and statistical analysis (lower) showed the effects of TMEM209 knockdown on colony formation in MHCC-97H and Huh7 cells. **D** Representative images of EdU experiments (left) and statistical analysis (right) showed the cell proliferation efficiency in the indicated groups. **E** The effects of TMEM209 knockdown on migration (upper) and invasion (lower) were evaluated by the Transwell assays. (Scale bar = 200 μm). **F** Wound healing assays were performed to detect the effects of TMEM209 knockdown on the motility of MHCC-97H and Huh7 cells. **G** Protein levels of PCNA, Cyclin D1, E-CAD, N-CAD, and vimentin and corresponding statistical results for the indicated groups. The results are presented as the mean ± SD of three independent experiments. n.s. indicates no significant difference. *P < 0.05, **P < 0.01, ***P < 0.001.

Subsequently, we determined the effects of TMEM209 on cell phenotypes in two HCC cell lines, namely MHCC-97H and Huh7. To this end, we knocked down the TMEM209 using shRNA, verified by western blotting and qPCR (Fig. 2A). Cells with an effective TMEM209 knockdown (sh-TMEM209#3) and control cells (sh-NC) were selected for subsequent experiments. CCK-8, colony formation, and EdU assays were performed to identify the role of TMEM209 in regulating the proliferative capacity of HCC, and the results indicated that TMEM209 downregulation significantly inhibited the viability of both cell lines (Fig. 2B–D). Subsequently, we assessed the effects of TMEM209 on cell motility. Transwell assays showed that knocking down TMEM209 significantly attenuated the migration and invasion of MHCC-97H/Huh7 cells (Fig. 2E). Similarly, wound healing assays confirmed a remarkable suppression of cell motility after knocking down TMEM209 (Fig. 2F). Furthermore, certain crucial proteins related to proliferation (PCNA, cyclin-D1) and EMT (E-CAD, N-CAD, vimentin) were assayed, and the results indicated that the knockdown of TMEM209 inhibited the proliferation and metastasis of HCC cells by abrogating the expression of proliferation-associated proteins and EMT progression (Fig. 2G). HCC cells overexpressing TMEM209 showed enhanced proliferation, migration, invasion and EMT (Fig. S2). Therefore, we concluded that TMEM209 promoted the proliferation and metastasis of HCC in vitro.

### TMEM209 augments the growth and metastasis of HCC cells through the Wnt/β-catenin signaling pathway

**Fig. 3:**
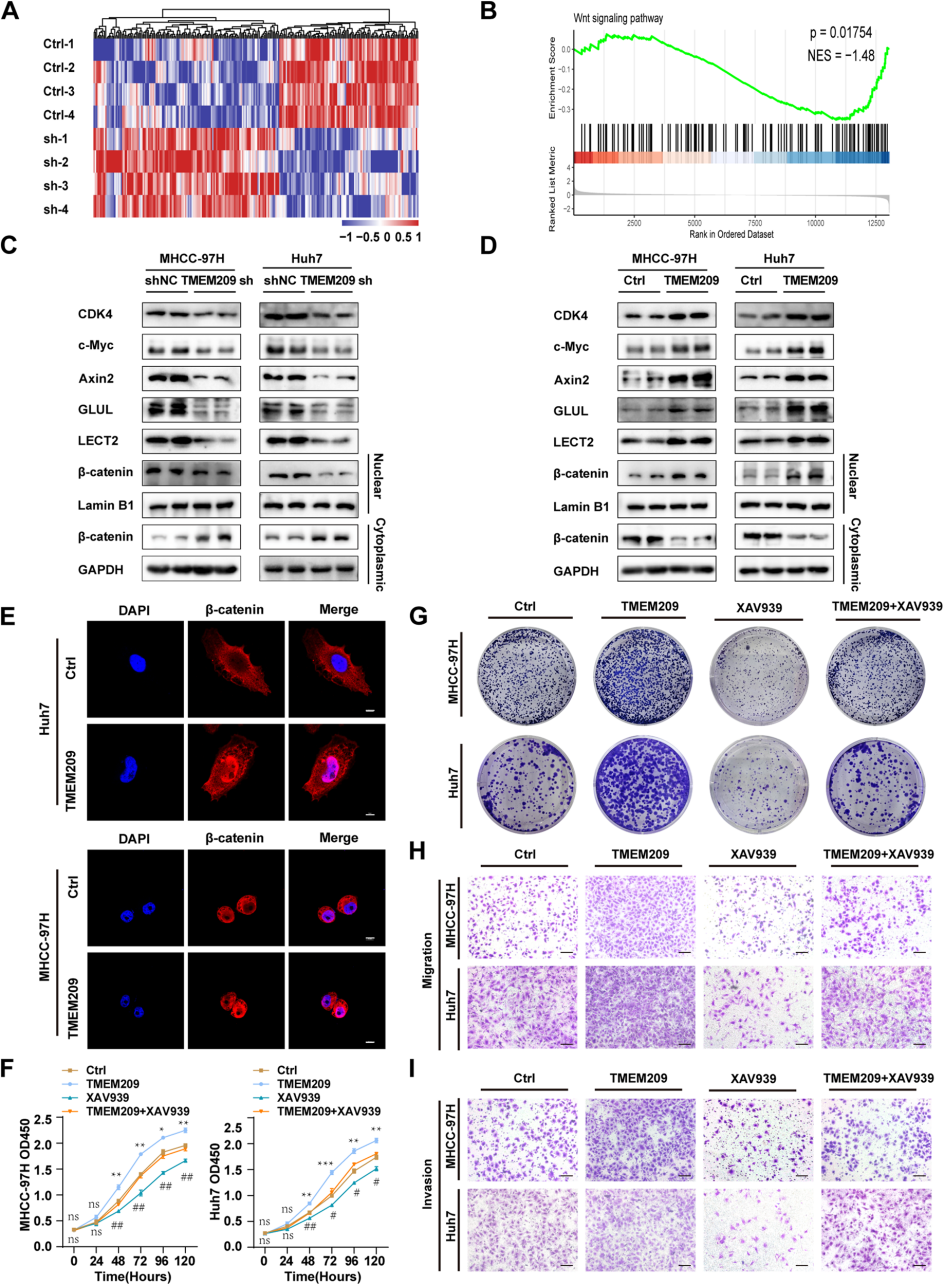
TMEM209 promotes the growth and metastasis of HCC cells through Wnt/β-catenin signaling pathway. **A** Heatmaps showing the differential expression of genes (Fold change > 1) in TMEM209 knocked down MHCC-97H cells and the control group. The expression levels values are shown as log2-transformed values. **B** GSEA revealed a significant enrichment of Wnt/β-catenin signaling pathway. Western blot for detecting the protein levels of c-Myc, CDK4, Axin2, GLUL, LECT2, and nuclear and cytoplasmic contents of β-catenin in TMEM209 knocked down (**C**) and overexpressing (**D**) MHCC-97H/Huh7 cells. **E** IF assays showing the effects of TMEM209 overexpression on the nuclear localization of β-catenin. (Scale bar = 10 μm). CCK-8 assays (**F**) and colony formation assays (**G**) showed the proliferation efficiency in the control group (Ctrl), TMEM209 overexpression group (TMEM209), XAV939(10 mM) treatment group (XAV939), and TMEM209 overexpression with XAV939 treatment group (TMEM209 + XAV939) of MHCC-97H and Huh7 cells. Representative images of migration (**H**) and invasion (**I**) in the Ctrl group, TMEM209 group, XAV939 group, and TMEM209 + XAV939 group by Transwell assays in MHCC-97H/Huh7 cells. (Scale bar = 200 μm). Note: XAV939 is a well-known inhibitor to block β-catenin activation. The results are presented as the mean ± SD of three independent experiments. *Indicates a significant difference between the Ctrl and TMEM209 groups. ^#^Indicates a significant difference between the TMEM209 and TMEM209 + XAV939 groups. n.s. indicates no significant difference. *P < 0.05, **P < 0.01, ***P < 0.001, ^#^*P* < 0.05, ^##^*P* < 0.01, ^###^*P* < 0.001.

After determining the functions of TMEM209 in HCC cells, we analyzed the underlying signaling pathway regulated by TMEM209. To this end, we performed RNA sequencing (Fig. 3A) and gene set enrichment analysis (GSEA) in TMEM209 knockdown of MHCC-97H cells. The results indicated that the Wnt/β-catenin signaling pathway was strongly associated with TMEM209 expression (Fig. 3B). Surprisingly, knocking down TMEM209 or overexpressing TMEM209 did not affect the protein levels of total β-catenin, total phosphorylated β-catenin (p-β-catenin-S675, p-β-catenin-S552, p-β-catenin-S33/37/T41), and total non-phosphor-β-catenin (non-p-β-catenin-S33/37/T41) (Fig. S3A, B). Generally, β-catenin exerts its functions after translocating to the nucleus. Therefore, we assessed the protein levels of β-catenin in the nuclear and cytoplasmic fractions. Consistent with our expectation, silencing TMEM209 inhibited the nuclear aggregation of β-catenin and promoted its accumulation in the cytoplasm (Fig. 3C). Additionally, levels of c-Myc, CDK4, Axin2, GLUL, and LECT2, critical downstream proteins of the Wnt/β-catenin signaling pathway, were decreased in TMEM209 knocked down cells (Fig. 3C). Furthermore, overexpression of TMEM209 yielded opposite results (Fig. 3D). Immunofuorescence (IF) staining supported the observed changes in β-catenin distribution (Figs. 3E and S4). Owing to previous research indicated that the mutation of β-catenin associated with the progression of tumor, we therefore investigated the relationship of TMEM209 expression and β-catenin’s mutation by analyzing TCGA data. The results showed no significant correlation between β-catenin’s mutation and TMEM209 expression (*P* = 0.07, Supplement table 1). Therefore, our findings suggested that TMEM209 promoted the nuclear translocation of β-catenin and activation of the Wnt/β-catenin pathway.

Subsequently, we conducted functional rescue experiments to check the involvement of TMEM209 through the Wnt/β-catenin signaling pathway. A well-known small-molecule inhibitor XAV939 was introduced to block the Wnt/β-catenin pathway [30, 31]. CCK-8 (Fig. 3F) and colony formation (Figs. 3G and S5A) assays indicated that XAV939 significantly inhibited TMEM209-stimulated cell proliferation. Furthermore, Transwell assays showed that XAV939 impaired the migration (Figs. 3H and S5B) and invasion (Figs. 3I, and S5C) abilities induced by TMEM209 overexpression. Therefore, TMEM209 could promote the proliferation and metastasis of HCC cells by activating the Wnt/β-catenin signaling pathway.

### TMEM209 interacts with KPNB1 and enhances its protein levels

**Fig. 4:**
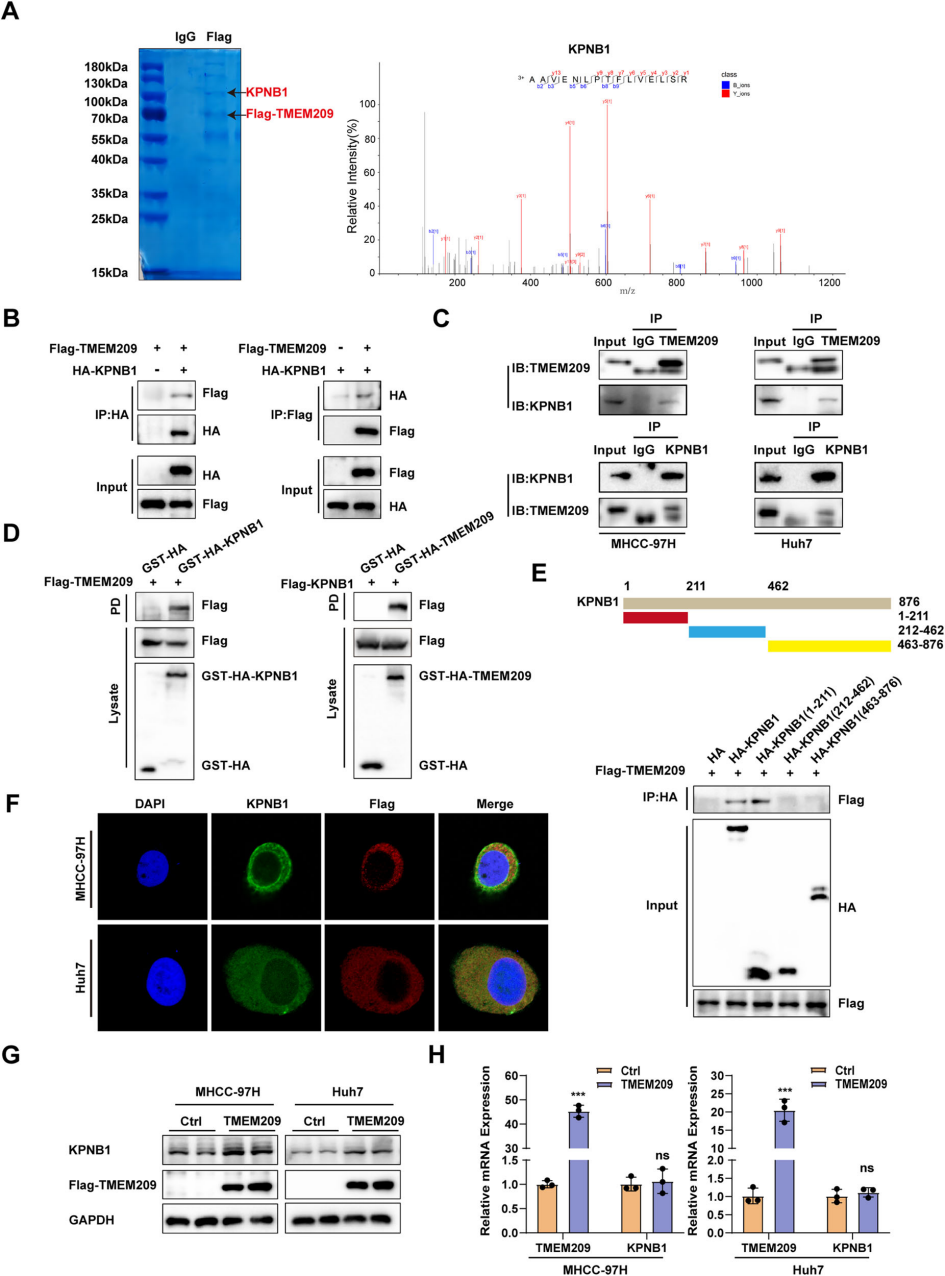
TMEM209 interacts with KPNB1 and enhances its protein expression. **A** Flag-TMEM209 overexpressing MHCC-97H cells were immunoprecipitated with a Flag or IgG antibody, followed by Coomassie brilliant blue staining of the 10% SDS-PAGE gel and mass spectrometric analysis. **B** Coimmunoprecipitation (Co-IP) assays for the interaction of TMEM209 and KPNB1 in HEK-293T cells transfected with Flag-TMEM209 or HA-KPNB1, alone or simultaneously. **C** Co-IP assays indicated the interaction between KPNB1 and TMEM209 in wild-type MHCC-97H and Huh7 cells. **D** The GST pulldown assays suggested a direct binding of TMEM209 and KPNB1 in HEK-293T cells. **E** Co-IP and western blot assays showed the binding region of KPNB1 with TMEM209 in HEK-293T cells. **F** IF staining reflected the colocalization of KPNB1 with TMEM209 in MHCC-97H/Huh7 cells. **G** Western blot analysis for protein levels of KPNB1 in MHCC-97H/Huh7 cells with TMEM209 overexpression. **H** The qPCR assays showed the mRNA levels of KPNB1 in TMEM209 overexpressing MHCC-97H/Huh7 cells and their corresponding controls. The results are presented as the mean ± SD of three independent experiments. n.s. indicates no significant difference. *P < 0.05, **P < 0.01, ***P < 0.001.

To further elucidate how TMEM209 activated the Wnt/β-catenin signaling pathway, we immunoprecipitated cell lysates with Flag antibody in Flag-TMEM209 overexpressed MHCC-97H cells, followed by mass spectrometric analysis. Additionally, we predicted a protein-protein interaction network for TMEM209 using the STRING database. Mass spectrometric and bioinformatic analyses collectively indicated KPNB1 as a potential protein interactor of TMEM209 (Figs. 4A and S6). Exogenous co-immunoprecipitation (Co-IP) assays confirmed that TMEM209 coimmunoprecipitated with KPNB1 and vice versa (Fig. 4B). Additionally, endogenous Co-IP assays confirmed this interaction (Fig. 4C), and GST-pull-down experiments suggested a direct interaction between TMEM209 and KPNB1 (Fig. 4D). KPNB1 is featured with an N-terminal domain and several repeat regions. The 1-211 residues of KPNB1 comprise the N-terminal domain and several HEAT regions. The 211-462 residues of KPNB1 possess regions interacting with RPL23A or binding to IAB or Ran-GTP. Other domains of KPNB1 incorporate several repeat regions. Further IP mapping assays indicated that the 1-211 residues of KPNB1 constituted the main fragment responsible for binding to TMEM209 (Fig. 4E). IF staining of MHCC-97H and Huh7 cells was performed to illustrate the results visually, which showed the co-localization of KPNB1 and TMEM209 in the cytoplasm (Fig. 4F).

Subsequently, we analyzed the regulatory relationship between KPNB1 and TMEM209. IHC analysis indicated that both TMEM209 and KPNB1 were upregulated in HCC tissues, even within the same position in the section (Fig. S7A). Western blotting indicated that TMEM209 overexpression significantly increased the protein levels of KPNB1, and silencing TMEM209 decreased them correspondingly (Figs. 4G and S7B). Contrary to our expectations, overexpression or knockdown of TMEM209 did not cause significant alterations in the mRNA levels of KPNB1 (Figs. 4H and S7C). Based on the above results, we found that TMEM209 interacted with KPNB1 and promoted its protein expression.

### TMEM209 promotes KPNB1’s stability by competitively inhibiting RCHY1-mediated ubiquitination

**Fig. 5:**
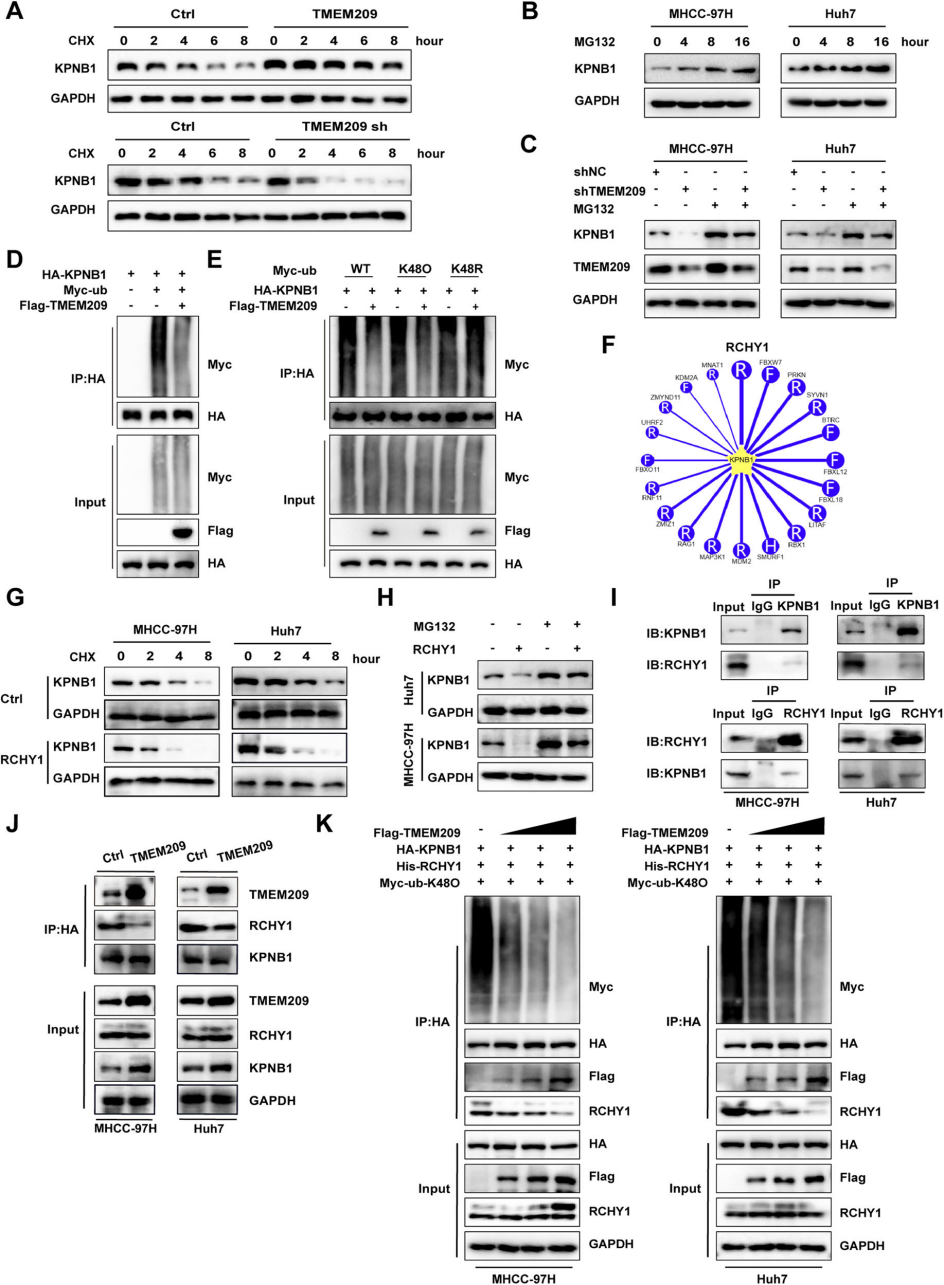
TMEM209 promotes the stability of KPNB1 by inhibiting RCHY1-mediated ubiquitination. **A** Western blot assay showing KPNB1 protein expression in MHCC-97H cells with TMEM209 overexpression or knockdown, under treatment with synthesis inhibitor, cycloheximide (CHX, 50 µg/ml) for 0, 2, 4, 6, and 8 h. **B** Representative western blot results for KPNB1 protein in MHCC-97H/Huh7 cells treated with MG132 (10 µM) for 0, 4, 8, and 16 h. **C** MHCC-97H/Huh7 cells stably expressing shNC and shTMEM209 plasmids were treated with or without MG132 (10 µM) for 24 h and then subjected to immunoblotting analysis. **D** Western blot results of ubiquitinated KPNB1 and total KPNB1 levels in HEK-293T cells transfected with the TMEM209 or control plasmid. **E** Western blot results of the expression of KPNB1 ubiquitination in HEK-293T cells transfected with the indicated plasmid. **F** E3 ubiquitin ligases mediating the degradation of KPNB1 were predicted by UbiBrowser analysis. **G** Western blotting showing the protein level of KPNB1 in MHCC-97H/Huh7 cells transfected with RCHY1 and subjected to CHX (50 µg/ml) treatment. **H** Western blot assay showing the expression of KPNB1 in MHCC-97H/Huh7 cells with RCHY1 overexpression or MG132 treatment, alone or simultaneously. **I** Co-IP assay indicated the interaction between KPNB1 and RCHY1 in MHCC-97H/Huh7 cells. **J** Immunoprecipitation and western blot assay were performed to analyze the binding affinity of KPNB1, TMEM209 and RCHY1 in MHCC-97H/Huh7 cells with overexpressing TMEM209. **K** Western blotting showed the effects of TMEM209 on the affinity of KPNB1 and RCHY1 in cells transfected with increasing doses of TMEM209.

Next, we analyzed the mechanism underlying the regulation of protein expression of KPNB1 through TMEM209. Cycloheximide (CHX, a protein synthesis blocking agent) was used to block protein synthesis in cells and we found the levels of KPNB1 protein significantly decreased after 4 h of CHX treatment. Additionally, our experiments suggested that overexpression and knockdown of TMEM209 slowed and accelerated the degradation of KPNB1, respectively (Figs. 5A and S8A). Then, proteasome inhibitor MG132 and lysosomal inhibitor chloroquine were introduced to inhibit protein degradation in MHCC-97H and Huh7 cells. Western blot analysis indicated that only MG132 attenuated the degradation of the KPNB1 protein (Figs. 5B and S8B). Importantly, pharmacological inhibition of proteasome by MG132 rescued KPNB1 downregulation induced by TMEM209 knockdown, suggesting the critical role of the ubiquitin-proteasome pathway in KPNB1 degradation (Fig. 5C). Subsequently, our experiments demonstrated that TMEM209 inhibited the K48-linked ubiquitination of KPNB1 (Fig. 5D, E). Collectively, our findings suggested that TMEM209 promoted the stability of KPNB1 by inhibiting its ubiquitination.

Typically, E3 ubiquitin ligases are essential in ubiquitination as they recognize target proteins. Therefore, we predicted that the E3 ubiquitin ligases affected KPNB1 and found that RCHY1 was the most crucial E3 ubiquitin ligase involved in KPNB1 ubiquitination (Fig. 5F). In fact, our findings demonstrated that overexpression of RCHY1 accelerated the degradation of KPNB1 (Fig. 5G) and revealed that MG132 could partially rescue the enhancing effects of RCHY1 on KPNB1 degradation (Fig. 5H). In addition, we confirmed the interaction of RCHY1 with KPNB1 (Fig. 5I) and further identified that RCHY1 promoted the ubiquitination of KPNB1 in HEK-293T cells (Fig. S9A, B). Subsequently, we analyzed the effects of TMEM209 on the binding of KPNB1 and RCHY1. Co-IP experiments indicated that TMEM209 overexpression attenuated the binding affinity of KPNB1 to RCHY1 (Fig. 5J). Interestingly, we observed that TMEM209 attenuated the binding affinity of KPNB1 to RCHY1 in a dose-dependent manner, along with the K48-linked ubiquitination of KPNB1 and boosted the KPNB1 protein levels in MHCC-97H and Huh7 cells (Fig. 5K). All in all, our experiments confirmed that TMEM209 stabilized KPNB1 by competitively attenuating its ubiquitination by RCHY1.

### KPNB1 promotes the proliferation and metastasis of HCC cells through Wnt/β-catenin signaling pathway

**Fig. 6:**
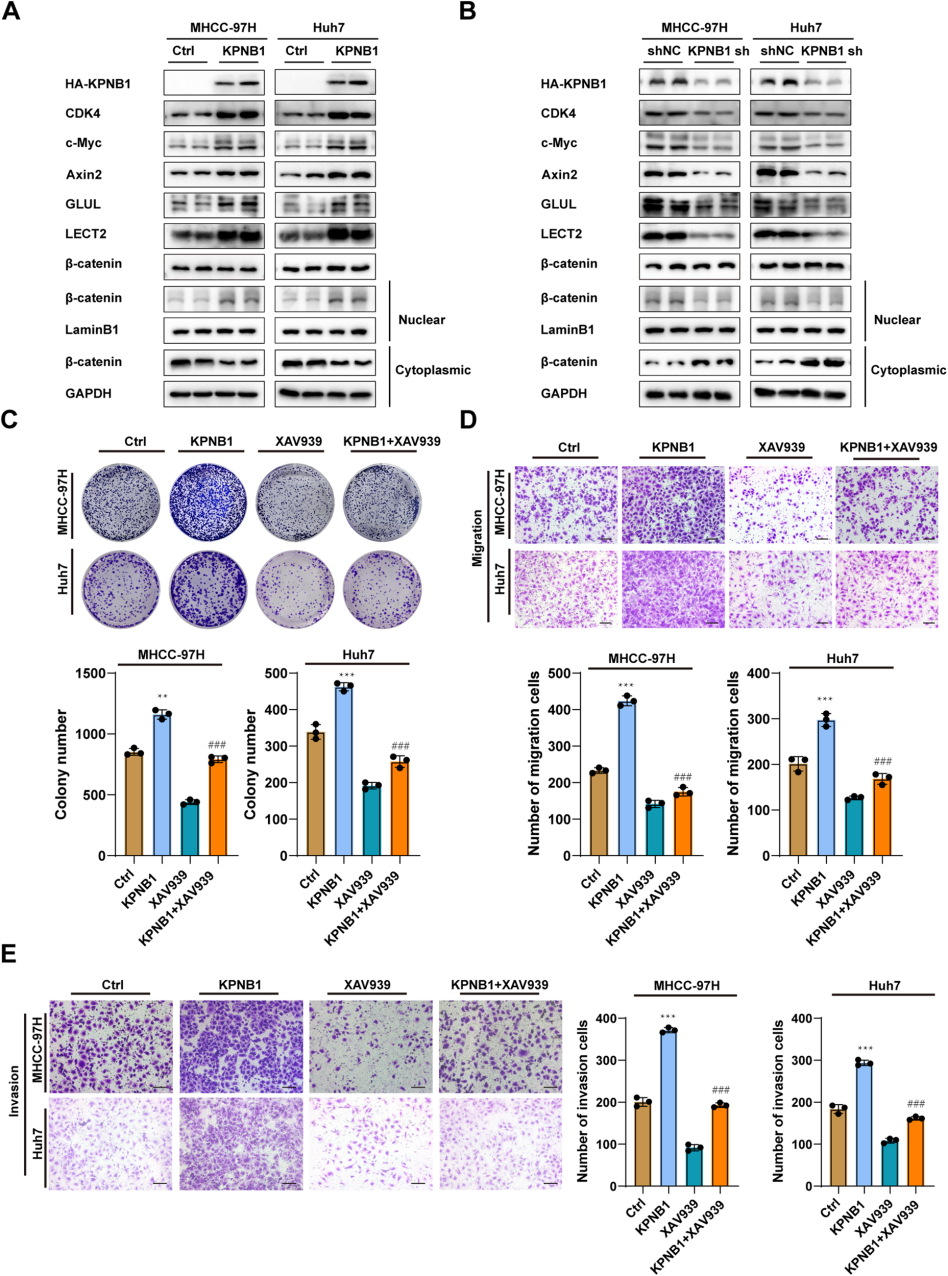
KPNB1 promotes the proliferation and metastasis of cells through Wnt/β-catenin signaling. Total β-catenin, cytoplasmic and nuclear β-catenin, c-Myc, CDK4, Axin2, GLUL and LECT2 in KPNB1 overexpressing (**A**) or knocked down (**B**) MHCC-97H/Huh7 cells. **C** Colony formation assay in MHCC-97H and Huh7 cells in the indicated groups. Transwell migration assay (**D**) and Transwell invasion assay (**E**) in MHCC-97H and Huh7 cells in the indicated groups. (Scale bar = 200 μm). The results are presented as the mean ± SD of three independent experiments. *Indicates a significant difference between the Ctrl and KPNB1 groups. ^#^Indicates a significant difference between the KPNB1 and KPNB1 + XAV939 groups. n.s. indicates no significant difference. **P < 0.01, ***P < 0.001; ^#^*P* <0.05, ^##^*P* < 0.01, ^###^*P* < 0.001.

After identifying the regulatory relationship of KPNB1 with TMEM209, we investigated the effects of KPNB1 in the Wnt/β-catenin signaling pathway and its potential function in HCC. We observed that overexpression of KPNB1 triggered the increasing levels of typical Wnt/β-catenin pathway-related factors (β-catenin in nucleus, CDK4, c-Myc, Axin2, GLUL and LECT2), while KPNB1 silencing yielded opposite trends (Fig. 6A, B). Furthermore, the inhibition of the Wnt/β-catenin signaling pathway notably rescued the increased proliferation and metastasis induced by KPNB1 overexpression (Fig. 6C–E). Therefore, we confirmed that KPNB1 accelerated the progression of HCC by activating the Wnt/β-catenin pathway.

### TMEM209 promotes the proliferation and metastasis of HCC depending on KPNB1 in vitro and in vivo

**Fig. 7:**
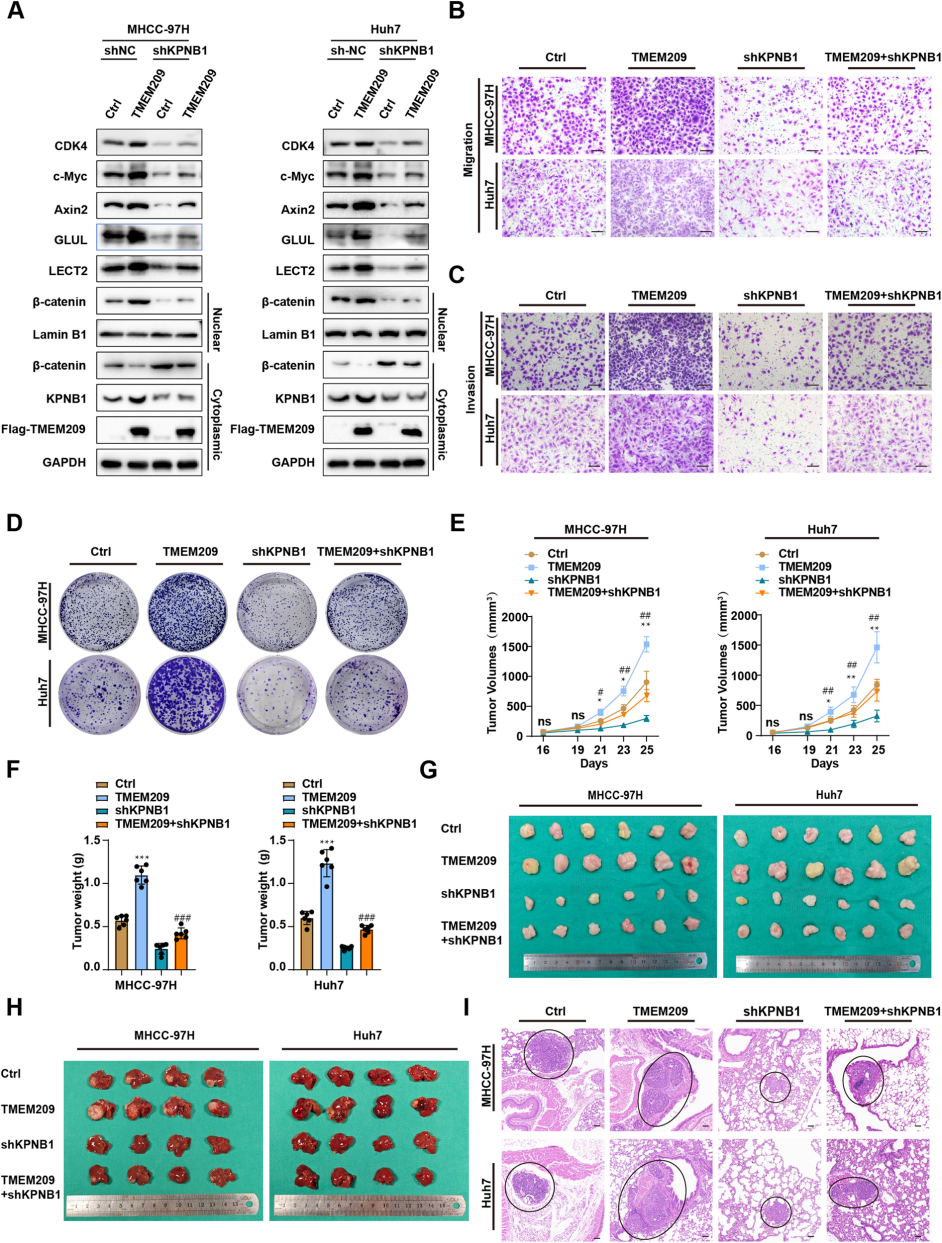
TMEM209 promotes the progression of HCC dependent on KPNB1. **A** Western blot analysis showing the protein levels of TMEM209, KPNB1, nuclear and cytoplasmic β-catenin, c-Myc, CDK4, Axin2, GLUL and LECT2 in MHCC-97H and Huh7 cells with TMEM209 overexpression and KPNB1 knockdown, alone or simultaneously. Transwell migration (**B**), invasion (**C**) assays and colony formation (**D**) assays were performed in MHCC-97H and Huh7 cells with TMEM209 overexpression and KPNB1 knockdown, alone or simultaneously. (Scale bar = 200 μm). The tumor volumes (**E**) of mice (n = 6) were measured on indicated days after MHCC-97H or Huh cells subcutaneous injection. Tumor weight (**F**) and images (**G**) from xenografted mice (n = 6) subcutaneous injection MHCC-97H or Huh cells in the indicated groups. **H** Tumor images from orthotopic transplantation tumor model of the liver (n = 4) in the indicated groups. **I** Lung tissues from the mice in indicated groups (n = 4) were stained with HE (Scale bar = 50 μm). The results are presented as the mean ± SD of three independent experiments. *Indicates a significant difference between the Ctrl and TMEM209 groups. ^#^Indicates a significant difference between the TMEM209 and TMEM209 + shKPNB1 groups. n.s. indicates no significant difference. *P < 0.05, **P < 0.01, ***P < 0.001; ^#^*P* < 0.05, ^##^*P* < 0.01, ^###^*P* < 0.001.

Next, we infected TMEM209 overexpressing cells with KPNB1-sh lentivirus (shKPNB1) or control lentivirus (Ctrl), respectively. Western blot analysis showed that KPNB1 knockdown blocked TMEM209-induced nuclear accumulation of β-catenin and the activation of CDK4, c-Myc, Axin2, GLUL, and LECT2 (Fig. 7A). Transwell assays suggested that the enhancing effects of TMEM209 on cell motility were suppressed (Figs. 7B, C and S10A). Similarly, colony formation assays showed the abrogation of TMEM209-induced cell colony formation by KPNB1-sh lentivirus transfection (Figs. 7D and S10B).

Finally, we conducted in vivo experiments. Control cells (Ctrl), TMEM209-overexpressing cells (TMEM209), KPNB1-silenced cells (shKPNB1), and cells with TMEM209 overexpression and KPNB1 silencing (TMEM209+shKPNB1) were injected into subcutaneous, tail vein or liver of BALB/c nude mice. In the subcutaneous tumor model, the tumor volume and weight in the TMEM209 group were higher than those of the Ctrl and TMEM209+shKPNB1 groups (Fig. 7E, F, n = 6). Importantly, silencing of KPNB1 slowed the tumor development induced by TMEM209 (Fig. 7E, F, n = 6). Tumor images visually reflected the size and weight difference of tumors in each group (Fig. 7G, n = 6). Similarity, the tumor images of orthotopic transplantation tumor model and haematoxylin & eosin (HE) staining of lung tissues from lung metastasis model indicated parallel results (Fig. 7H–I, Fig. S10C, n = 4). Therefore, we confirmed that the role of TMEM209 in HCC was dependent on KPNB1.

## Discussion

Previous findings have indicated that TMEMs exert pro-oncogenic effects in cancer development by regulating nucleoplasmic transport, chromatin dynamics, and cancer-related genes or pathways. To the best of our knowledge, TMEM209 has been implicated in promoting lung cancer growth and lymphoma [11, 12]. Intriguingly, it is a potential biomarker for drug evaluation [32]. However, the precise role of TMEM209 in HCC remains unclear. Our investigation revealed elevated TMEM209 levels in HCC tissues, and patients with high TMEM209 expression had poor prognosis. Moreover, TMEM209 demonstrated a pro-proliferative and metastatic role in HCC in vitro and in vivo. Consequently, our findings underscore TMEM209 as a critical oncogene, promising biomarker for prognostic evaluation, and treatment target for HCC.

The Wnt/β-catenin signaling pathway is typically activated in HCC and exerts oncogenic effects during its progression. Our study, GSEA, revealed that the pro-oncogenic effects of TMEM209 may be mediated through the Wnt/β-catenin signaling pathway. Earlier studies implied that TMEM48 and TMEM17, members of the TMEM family, promoted the progression of cervical cancer and breast cancer by enhancing the protein expression of β-catenin [7, 33]. Nonetheless, we did not observe any change in the total protein expression of β-catenin in HCC cells with TMEM209 overexpression or knockdown. As β-catenin functions as a transcription factor, nuclear translocation of β-catenin is crucial for its biological function and the activation of Wnt/β-catenin signaling pathway. Previous studies highlighted that nuclear translocation of β-catenin significantly promoted HCC progression [31, 34]. Therefore, we speculated whether TMEM209 affected the localization of β-catenin to regulate the Wnt/β-catenin signaling pathway. Our results verified the hypothesis that TMEM209 activated the Wnt/β-catenin signaling pathway by enhancing the nuclear translocation of β-catenin rather than its expression. Blocking the Wnt/β-catenin signaling pathway confirmed that TMEM209 augmented the proliferation and metastasis of HCC through Wnt/β-catenin signaling.

To decipher the contribution of TMEM209 to carcinogenesis and Wnt/β-catenin signaling, mass spectrometry analysis identified KPNB1 as a potential protein interacting with TMEM209. We confirmed, for the first time, that KPNB1 directly interacted with TMEM209. As a member of the importin family, KPNB1 facilitates the nuclear import of key proteins, including p65 and β-catenin, activating corresponding signaling pathways implicated in the progression of several malignancies [15, 35]. Consistent with previous findings, our results showed that KPNB1 promoted the proliferation and metastasis of HCC cells [15, 36]. Similar to the study on glioma, the role of KPNB1 in HCC was found to be dependent on the activation of Wnt/β-catenin signaling.

We confirmed that TMEM209 only enhanced the protein levels of KPNB1. Specifically, KPNB1’s regulation by TMEM209 occurred during protein degradation rather than during protein synthesis. Commonly, protein degradation and stability regulation are mediated by autophagy or ubiquitination, and few studies have focused on the roles of TMEMs in maintaining protein stability. To the best of our knowledge, TMEM63A stabilized oncogene DER1 by preventing its autophagic degradation and TMEM97 inhibited the ubiquitination degradation of β-catenin [37, 38]. Interestingly, previous study indicated that TMEM209 stabilized NUP205 in the protein level in lung cancer cells; however, a detailed understanding of its mechanism was lacking. Our experiments confirmed that the proteasome inhibitor MG132, rather than chloroquine, inhibited the degradation of KPNB1. Intriguingly, residues 1–211 of KPNB1 (the region of KPNB1 interacting with TMEM209) were present at multiple ubiquitination loci [39]. We reported for the first time that TMEM209 inhibited the ubiquitination and degradation of KPNB1.

**Fig. 8:**
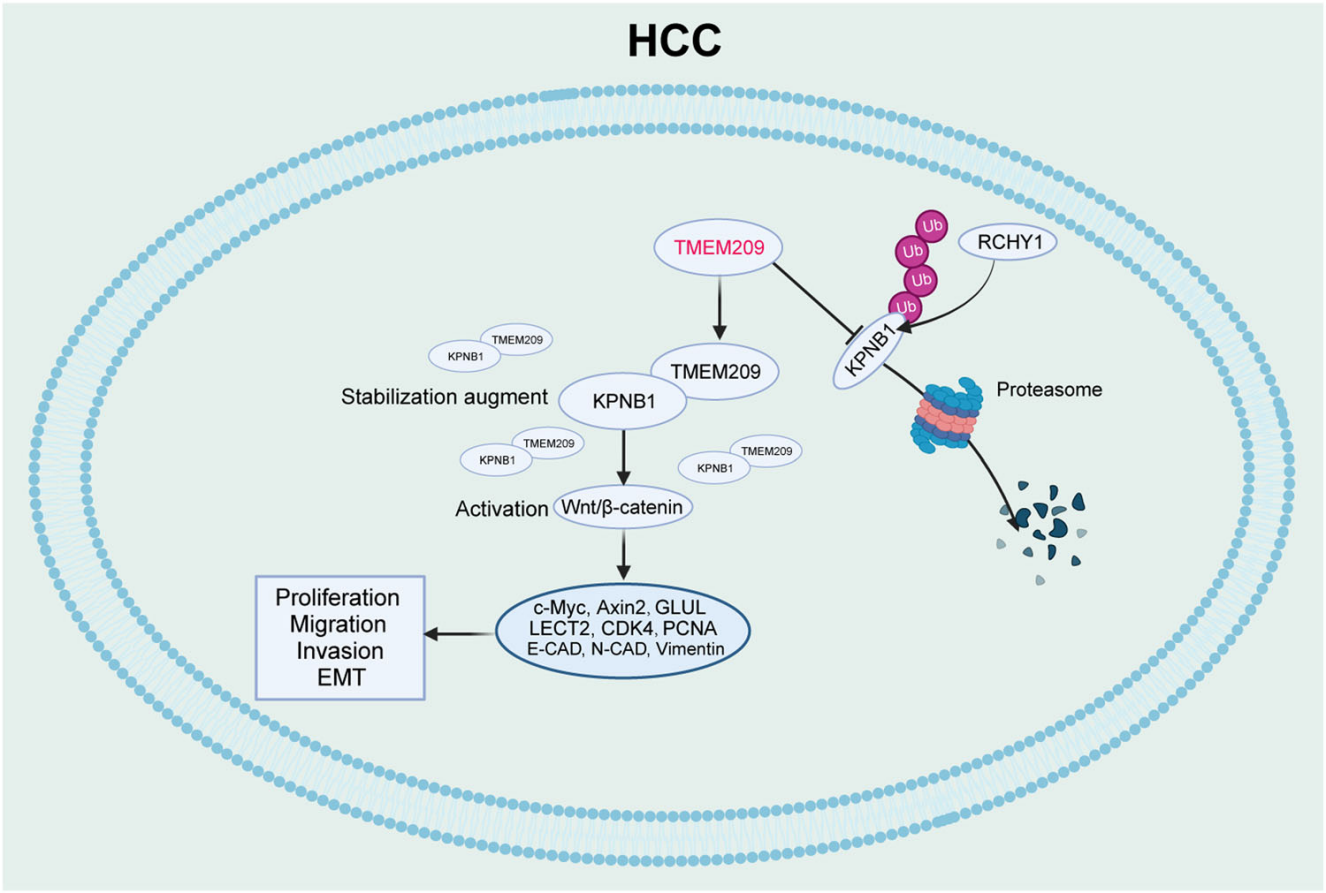
Patterns revealing the relationship between TMEM209, β-catenin, KPNB1 and RCHY1.

Generally, E3 ligases interact with the target protein, promoting their ubiquitination and subsequent degradation, while deubiquitinating enzymes exhibit the opposite effects. However, TMEM209 is not characterized as a deubiquitinating enzyme; therefore, existing evidence cannot explain our findings. E3 ligases and ubiquitination process are regulated or modified by various proteins [40, 41]. Accordingly, we proposed a hypothesis independent of a deubiquitinating enzyme wherein TMEM209 may inhibit the ubiquitination of KPNB1 by disturbing the interaction of KPNB1 with E3 ligase. The E3 ligase, RCHY1, regulates the ubiquitination of various oncogenes and tumor depressors, including HDAC2, p53, and twist, potentially affecting KPNB1 degradation [42–44]. Furthermore, our experiments confirmed that RCHY1 promoted the ubiquitination of KPNB1. As expected, we observed that TMEM209 stabilized KPNB1 by competitively inhibiting the binding of KPNB1 with RCHY1. Although the mechanism of competitive binding regulating protein functions is widely understood, it has been rarely investigated in ubiquitination and tumor progression. Interestingly, a recent study presented a similar regulatory mechanism in lung cancer, where plant homologous structural domain finger protein 23 (PHF23) enhanced the stability of alpha-actinin-4 (ACTN4) by inhibiting its interaction with E3 ligase [41]. This expands the role of non-deubiquitinating enzymes in regulating ubiquitination, contributing to understanding the mechanism of tumor progression in depth and identifying potential targets. Consequently, our findings propose a novel mechanism of HCC progression mediated by TMEM209, that is, TMEM209 activates Wnt/β-catenin signaling by preventing ubiquitination degradation of KPNB1 (Fig. 8). This regulatory axis sheds new light on the understanding of the malignant progression of HCC.

In conclusion, our findings revealed the role and clinical significance of TMEM209 and confirmed the crucial regulatory role of the TMEM209/KPNB1/Wnt/β-catenin axis in HCC progression. Our study indicated that TMEM209 may serve as a promising biomarker for prognostic evaluation, and targeting TMEM209 and its regulatory axis may act as a novel potential therapeutic strategy for treating patients with HCC.

## Materials and methods

### Patients

Clinical specimens were obtained from patients with pathologically confirmed HCC diagnosis at the First Affiliated Hospital of Zhengzhou University. All specimens were collected within 30 min post-hepatectomy and appropriately preserved. No patient underwent radiotherapy or chemotherapy prior to surgery. Patient information and outcomes have been followed up since 2018.

### Animal experiments

All protocols of animal experiments were approved by the Animal Care Committee of The First Affiliated Hospital of Zhengzhou University. BALB/c nude mice (4-weeks-old, weighing 15-18 g) were purchased from the Vital River Company (Beijing, China). Animals were fed and maintained under similar feeding conditions. Mice were randomly divided into various groups and experiments were performed by single-blind way. A xenograft tumor model was established by subcutaneously injecting 5 × 10^6^ cells in 200 μl phosphate-buffered saline (PBS). From 16 days, the sizes of the tumors in the animals were measured regularly. Tumor volume was calculated as follows: V = (length×width^2^)/2. Mice were sacrificed, and the tumors were dissected after 25 days. To establish lung metastasis models, 3 × 10^6^ cells in 200 μl PBS were injected into the tail vein. After 8 weeks, animals were sacrificed, and their lung tissues were collected and subjected to HE staining. For the orthotopic transplantation tumor model of the liver, 3 × 10^6^ cells in 30 μl Matrigel (354230, BD Biosciences, USA) diluted with PBS were injected into the liver. After 5 weeks, mice were euthanized, and their liver tissues were collected.

### Cell culture and plasmids

Huh7 and MHCC-97H cells were obtained from Icell Bioscience (Shanghai, China) and identified by short tandem repeat (STR) analysis. All cells were cultured following standardized procedures.

Small hairpin RNAs (shRNAs) targeting TMEM209 and KPNB1 were obtained from GenePharma (Shanghai, China). TMEM209 sequences with HA or FLAG tags were synthesized by Hanbio (Shanghai, China). Other plasmids constructs were synthesized by Tsingke (Suzhou, China). The shRNA sequences are presented in Supplementary Table 2.

### IHC and IF staining

IHC assays and scoring were conducted as described previously [45]. For IF staining, cells were first fixed with 4% paraformaldehyde, followed by permeabilized with (0.1%) Triton X-100 and incubated with corresponding primary antibodies at 4 °C overnight. Next, cells were incubated with fluorophore-conjugated secondary antibodies for 1 h at room temperature. Cell nuclei were stained with DAPI. The antibodies in experiments are presented in Supplementary Table 3.

### Western blot, IP, and ubiquitination assays

Total protein samples were extracted using RIPA lysis buffer (Beyotime Biotechnology) according to standard protocol. Nuclear and cytoplasmic proteins were fractionated and obtained following the manufacturer’s instructions (Beyotime Biotechnology). The samples were electrophoresed on sodium dodecyl sulfate-polyacrylamide gels. After transfer to PVDF membranes, they were incubated with the primary antibodies at 4 °C for 12 h, followed by incubation of secondary antibodies for 1 h at room temperature. ECL chemiluminescence imaging system (ImageQuant 800, USA) was used to detect protein signals. Lamin B1 was used as a normalization control for the nuclear proteins, while GAPDH served as an internal control for other proteins.

For IP assays, the cells were transfected with the corresponding plasmids and lysed using the IP lysis buffer (Beyotime Biotechnology). The partial lysates were separated as the input. Other lysates were incubated with the corresponding antibody-bound magnetic beads (Beyotime Biotechnology) at 4 °C for 12 h. After washing thrice, the proteins were eluted as IP for western blotting or mass spectrometry analysis. For ubiquitination assays, cells were initially lysed with IP lysis buffer. An additional 10% SDS was added and denatured at 95 °C. After ultrasonication, the relevant proteins were collected by IP and assayed by western blotting. The antibodies are presented in Supplementary Table 3.

### qPCR

Total RNA was extracted using the TRIzol reagent (Invitrogen). Subsequently, 1 μg RNA was used to synthesize cDNA according to the protocols of the cDNA Synthesis Kit (Vazyme). The qPCR was carried out with specific primers and the ChamQ SYBR qPCR Master Mix (Vazyme). The 2^-ΔΔCT^ method was employed to determine relative changes in the expression of target genes. Housekeeping gene GAPDH was used to normalize other genes. The primer sequences are presented in Supplementary Table 4.

### Cell growth and proliferation assays

Cells were seeded in 96-well plates at a density of 3000 cells/well and incubated with 10% CCK-8 reagent (Dojindo, Shanghai, China) for 1 h at 0, 24, 48, 72, and 96 h. Absorbance was measured at 450 nm on a microplate reader (Varioskan LUX, Thermo, USA). To assess colony formation, cells were inoculated into 6-well plates at a density of 1000 cells/well and cultured for 10–14 days. After fixing and staining, colonies comprising more than 50 cells were counted and imaged. EdU assay was performed using the EdU Kit (Beyotime Biotechnology) to assess cell proliferation following the manufacturer’s instructions.

### Wound healing assays, Transwell migration, and invasion assays

Approximately 10^6^ cells were inoculated into each well of 6-well plates. After 12 h, 10 µl plastic pipette tips were used to generate wounds. Subsequently, cells were cultured in DMEM without fetal bovine serum (FBS) for 24 h. Images were taken at 0 h and 24 h using a microscope (Olympus, Tokyo, Japan). The scratched area was determined to assess the migration ability of cells.

For migration assays, cells were seeded on the upper chamber of Transwell plates (3422; Corning, USA) at a density of 5 × 10^4^ cells/well in DMEM without FBS. The lower chamber contained DMEM with 10% FBS as the stimulatory agent. After 24–72 h, infiltrating cells were fixed with paraformaldehyde and stained using crystal violet. Images were obtained using a microscope (Olympus, Tokyo, Japan). For invasion assays, the upper chamber was covered with Matrigel (354230, BD Biosciences, USA) before inoculating cells. The remaining steps were the same as those for assessing migration.

### RNA sequencing and bioinformatics analysis

Total RNA from TMEM209 knocked-down MHCC-97H cells and control cells (n = 4) were extracted for sequence. RNA sequencing and data analysis were performed by Biomarker Technologies (http://www.biomarker.com.cn/). Expression and correlation analyses of TCGA data were performed using the gene expression profiling interactive analysis online program (http://gepia.cancer-pku.cn/) [46]. Survival analysis of the TCGA dataset was performed using the KM plotter online program (https://kmplot.com/analysis/) [47]. The E3 ubiquitin ligase-substrate interaction network was analyzed on UbiBrowser (http://ubibrowser.ncpsb.org) [48].

### Statistical analysis

Statistical analysis was conducted using the SPSS software (version 21.0; SPSS Inc., Chicago, IL, USA) and GraphPad PRISM (version 9). Data are presented as mean ± SD. Patient survival was compared by KM analysis and significance was assessed using log-rank tests. Differences between the two groups were analyzed using a two-tailed Student’s *t* test. Multiple group comparisons were performed using one-way analysis of variance or two-way analysis of variance. *P* < 0.05 indicated statistical significance.

## Supplementary information

SUPPLEMENTAL MATERIAL

Original western blot brands

## Data Availability

The authors declare that all the data generated or analyzed in this study are available within this article or supplementary information files.
