# Supplementary material for: TMEM209 promotes hepatocellular carcinoma progression by activating the Wnt/β-catenin signaling pathway through KPNB1 stabilization
Source: Cell Death Discov. 2024 Oct 16;10:438. doi: 10.1038/s41420-024-02207-9 (PMC11484822; doi:10.1038/s41420-024-02207-9)
Supplement: Supplementary file 1 — SUPPLEMENTAL MATERIAL [file 41420_2024_2207_MOESM1_ESM.pdf]

TMEM209 promotes hepatocellular carcinoma progression by activating the Wnt/β-catenin signalling pathway through KPNB1 stabilization

Haoran Fang[*1,2,3], Xiaoyi Shi[*1,3], Jie Gao[1,2,3], Zhiping Yan[1,3], Yun Wang[1], Yabin Chen[1,2,3], Jiacheng Zhang[1], Wenzhi Guo[1,2,3]

[1]Department of Hepatobiliary and Pancreatic Surgery, The First Affiliated Hospital of Zhengzhou University, Zhengzhou, China

[2]Henan Engineering & Research Center for Diagnosis and Treatment of Hepatobiliary and Pancreatic Surgical Diseases, Zhengzhou, China

[3]Henan Key Laboratory of Digestive Organ Transplantation, Zhengzhou, Henan, China.

[*]These authors contributed equally to this work

Correspondence:

Jiacheng Zhang

aster65@163.com

Wenzhi Guo

fccguowz@zzu.edu.cn

Table of content

Supplementary figures..2

Supplementary tables...9

Supplementary figures

Supplement figure 1

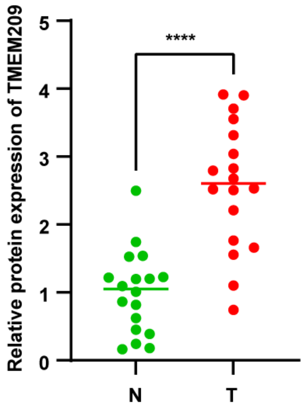

Fig. S1. Relative quantitative analysis of TMEM209 protein expression in Fig 1D. N = normal tissues, T = tumor tissues. ****$\boldsymbol{P} < 0.0001$.

Supplement figure 2

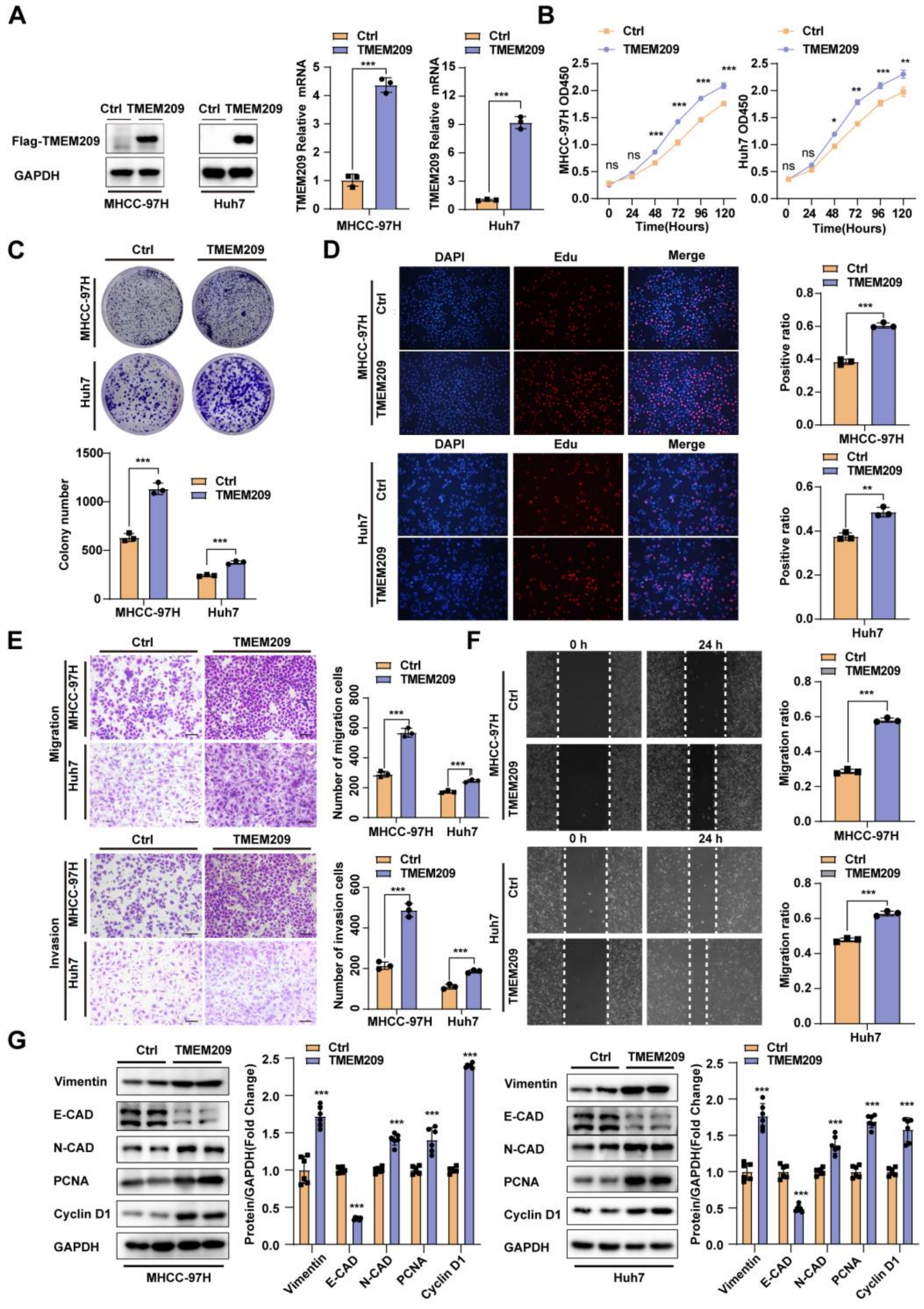

Fig. S2. Overexpression of TMEM209 promotes the proliferation and metastasis of HCC cells *in vitro*

A. Expression of TMEM209 in MHCC-97H and Huh7 cells was evaluated by western blot and qPCR. **B.** CCK-8 assays were performed to detect the effects of TMEM209 overexpression on the viability of MHCC-97H and Huh7

cells. **C.** Representative images (upper) and statistical results (lower) showed the effects of TMEM209 overexpression on colony formation in MHCC-97H and Huh7 cells. **D.** Representative images of EdU experiments (left) and statistics (right) showing the proliferation efficiency of MHCC-97H and Huh7 cells in the indicated groups. **E.** The effects of TMEM209 overexpression on migration (upper) and invasion (lower) in MHCC-97H and Huh7 cells were evaluated by Transwell assays. (Scale bar =200 μm). **F.** Wound healing assays were performed to assess the effects of TMEM209 overexpression on the motility of MHCC-97H and Huh7 cells. **G.** Protein levels of PCNA, Cyclin D1, E-CAD, N-CAD, and vimentin and corresponding statistical results for the indicated groups. The results are presented as the mean ± SD of three independent experiments. $^{*}\boldsymbol{P} < 0.05$, $^{**}\boldsymbol{P} < 0.01$, $^{***}\boldsymbol{P} < 0.001$.

Supplement figure 3

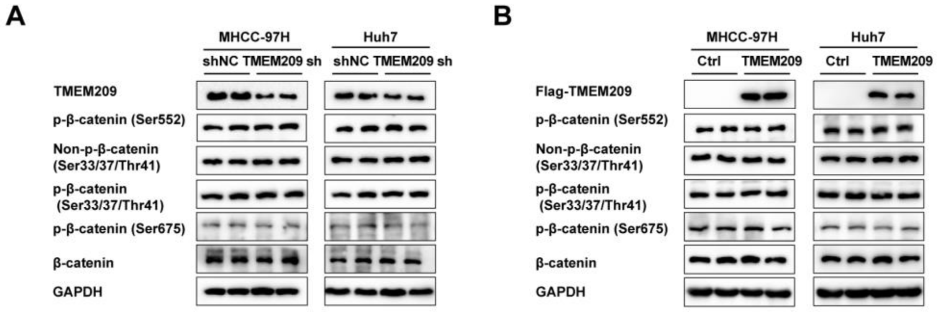

Fig. S3. TMEM209 does not affect the total protein levels and phosphorylation level of key molecules involving the Wnt/β-catenin signaling pathway

A. B. Total β-catenin, phosphorylated β-catenin (p-β-catenin-S675, p-β-catenin-S552, p-β-catenin-S33/37/T41) and non-phosphor-β-catenin (non p-β-catenin-S33/37/T41) levels were detected by western blot in TMEM209 knocked down (**A**) and overexpressing (**B**) MHCC-97H/Huh7 cells.

Supplement figure 4

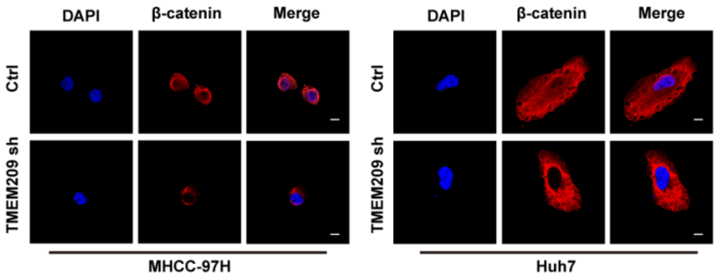

Fig. S4. Knockdown TMEM209 inhibits the aggregation of β-catenin in nucleus

IF assays showing the effects of TMEM209 knocked down on the nuclear localization of β-catenin. (Scale bar = 10 μm).

Supplement figure 5

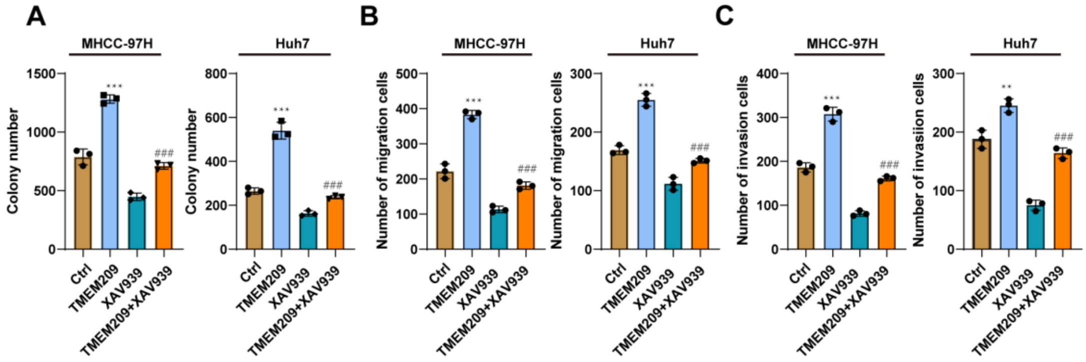

Fig. S5. TMEM209 promotes the proliferation and metastasis of HCC cells by the Wnt/β-catenin signalling pathway

A. The statistical plot of Fig. 3G showing that the number of colonies in the indicated group. **B.** The statistical plots of Fig. 3H, and Fig. 3I showing that the numbers of cell migration and invasion in the indicated group. The results are presented as the mean ± SD of three independent experiments. *Indicates a significant difference between the Ctrl and TMEM209 groups. $^{\#}$Indicates a significant difference between the TMEM209 and TMEM209+XAV939 groups. $^{**}\boldsymbol{P} < 0.01$, $^{***}\boldsymbol{P} < 0.001$; $^{\#\#}\boldsymbol{P} < 0.01$, $^{\#\#\#}\boldsymbol{P} < 0.001$.

Supplement figure 6

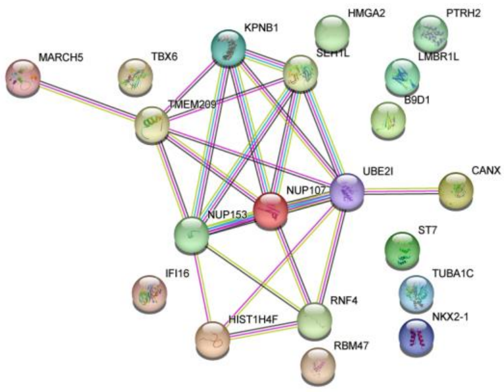

Fig. S6. Bioinformatic analysis predicts the potential proteins interacting with TMEM209

Protein-protein interaction (PPI) network based on the STRING database.

Supplement figure 7

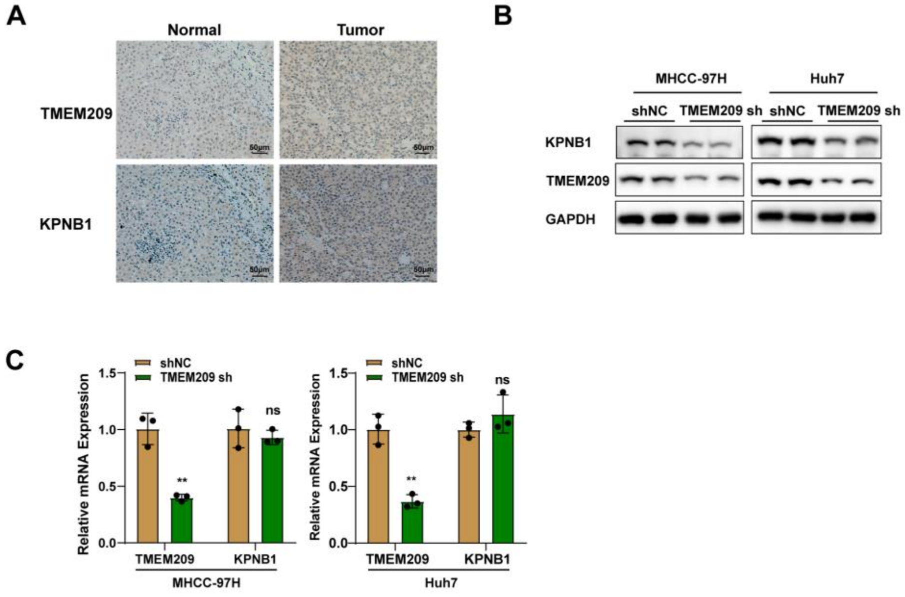

Fig. S7. TMEM209 enhances the expression of KPNB1 at the protein level rather than RNA level.

A. Representative IHC staining showing TMEM209 and KPNB1 protein levels at the same position in the tissue section (Scale bar = 50 μm). **B.** Western blot analysis for protein levels of KPNB1 in MHCC-97H/Huh7 cells with

TMEM209 knockdown. **C.** The qPCR assays showed the mRNA levels of KPNB1 in TMEM209 knocked down MHCC-97H/Huh7 cells and their corresponding controls. The results are presented as the mean ± SD of three independent experiments. n.s. indicates no significant difference. $^{*}\boldsymbol{P} < 0.05$, $^{**}\boldsymbol{P} < 0.01$.

Supplement figure 8

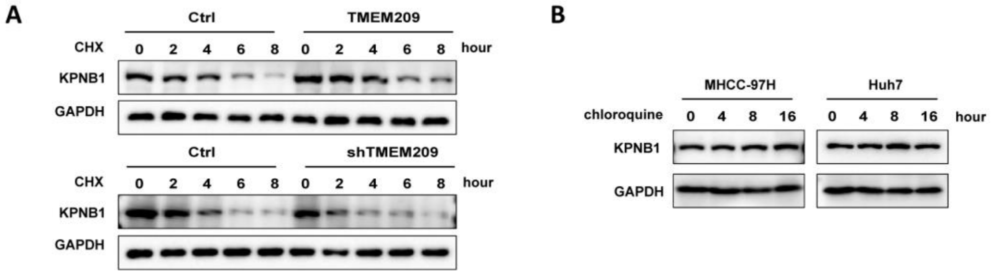

Fig. S8. TMEM209 augments the protein level of KPNB1 through post-translational modification.

A. Western blot showed KPNB1 protein expression in Huh7 cells with TMEM209 overexpression or knockdown, after treatment with the protein synthesis inhibitor, CHX (50 μg/ml) for 0, 2, 4, 6, and 8 h. **B.** Representative western blot analyses of KPNB1 protein levels in MHCC-97H/Huh7 cells treated with chloroquine (20 μM) for 0, 4, 8, and 16 h.

Supplement figure 9

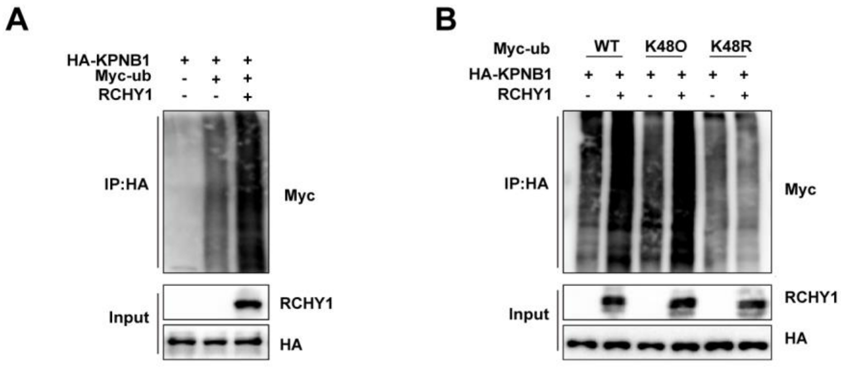

Fig. S9. RCHY1 promotes the K-48 linked ubiquitination of KPNB1.

A. Representative western blot results showed that RCHY1 promoted the ubiquitination of KPNB1 in HEK-293T

cells. **B.** Western blot analysis of the expression of ubiquitinated KPNB1 in HEK-293T cells transfected with the indicated plasmid.

Supplement figure 10

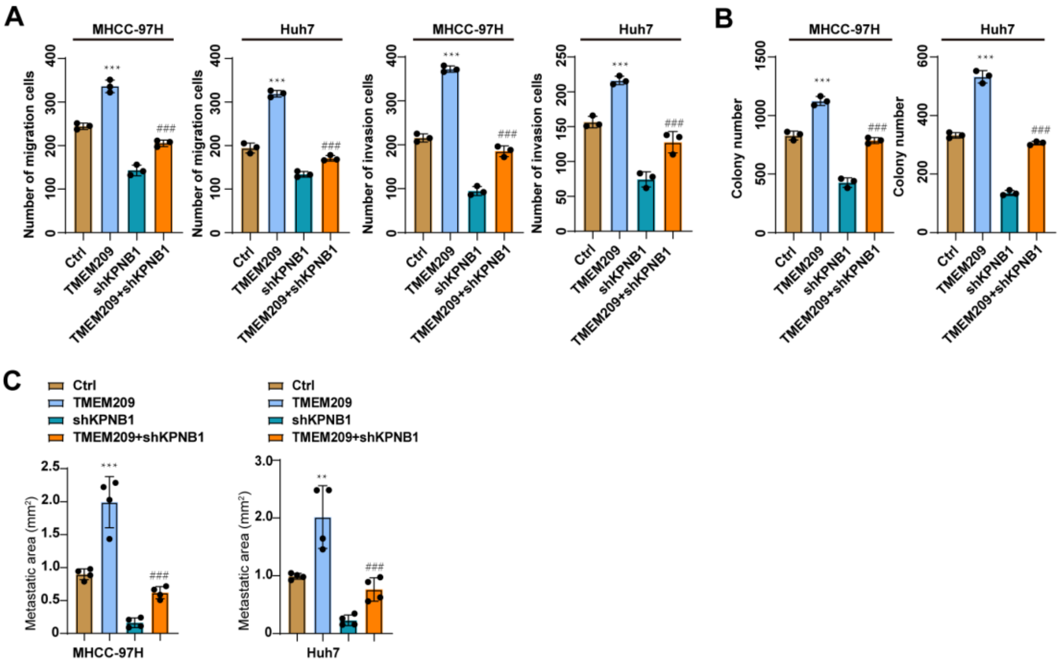

Fig. S10. TMEM209 promotes the progression of HCC dependent on KPNB1

A. B. The statistics of Fig. 7B-D indicating the number of migrating (**A**), invasion (**A**) cells, and the number of colonies (**B**) in the designated group. **C.** The statistics of Fig. 7I showing the metastatic area of tumor tissue in lung tissues in indicated group. The results are presented as the mean ± SD of three independent experiments. *Indicates a significant difference between the Ctrl and TMEM209 groups. #Indicates a significant difference between the TMEM209 and TMEM209+shKPNB1 groups. $^{*}\boldsymbol{P} < 0.05$, $^{**}\boldsymbol{P} < 0.01$, $^{***}\boldsymbol{P} < 0.001$; $^{\#\#}\boldsymbol{P} < 0.01$, $^{\#\#\#}\boldsymbol{P} < 0.001$.

Supplementary tables

Supplement table 1. The relationship of β-catenin's mutations and TMEM209 expression levels

	TMEM209 High	TMEM209 Low	*P* value
CTNNB1 Mutation	45	56	0.07
CTNNB1 Wildtype	145	118	

Supplement table 2. Sequence information of shRNA constructs

shRNA	Sequence (5'--3')
sh-TMEM209-1	GATCCGTCGCCCGTCAGTGGTTATAATTTCAAGAGAATTATAACCACTGACGGGCGATTTTTG
sh-TMEM209-2	GATCCGTGGGGACTCCTAAATGTATCTTTCAAGAGAAGATACATTTAGGAGTCCCCATTTTTG
sh-TMEM209-3	GATCCGATGGACAGCTAAATTTAGAAATTCAAGAGATTTCTAAATTTAGCTGTCCATTTTTTG
sh-NC	GATCCGTTCTCCGAACGTGTCACGTTTCAAGAGAACGTGACACGTTCGGAGAACTTTTTG
sh-KPNB1	CCGGGCTTGCTATTGATGCTAATGCCTCGAGGCATTAGCATCAATAGCAAGCTTTTTTG

Supplement table 3. Antibodies used in the study

Antibody	Cat No.	Manufacturer	Dilution
HA	51064-2-AP	proteintech	1:5000 (WB)
HA	66006-2-Ig	proteintech	1:5000 (WB)
Flag	80010-1-RR	proteintech	1:5000 (WB)<br>1:100 (IF)
Flag	66008-4-Ig	proteintech	1:5000 (WB)
TMEM209	sc-515223	santa cruz	1:200 (WB)<br>1:50 (IHC)
KPNB1	ab2811	abcam	1:3000 (WB)<br>1:200 (IF)

KPNB1	10077-1-AP	proteintech	1:1000 (WB)
RCHY1	5754	CST	1:1000 (WB)
GAPDH	60004-1-Ig	proteintech	1:10000 (WB)
β-catenin	AF0069	Beyotime	1:1000 (WB)<br>1:200 (IF)
p-β-catenin (Ser552)	AF5749	Beyotime	1:1000 (WB)
p-β-catenin (Ser675)	28853-1-AP	proteintech	1:1000 (WB)
Vimentin	10366-1-AP	proteintech	1:1000 (WB)
E-CAD	20874-1-AP	proteintech	1:1000 (WB)
N-CAD	22018-1-AP	proteintech	1:1000 (WB)
PCNA	10205-2-AP	proteintech	1:1000 (WB)
Cyclin D1	26939-1-AP	proteintech	1:100 (WB)
p-β-catenin (Ser33/37/Thr41)	9561T	CST	1:1000 (WB)
Non-p-β-catenin (Ser33/37/Thr41)	33893SF	CST	1:1000 (WB)
Lamin B1	12987-1-AP	proteintech	1:3000 (WB)
c-Myc	10828-1-AP	proteintech	1:1000 (WB)
Myc	M047-3	MBL	1:1000 (WB)
LECT2	ER61907	huabio	1:500 (WB)
Axin2	ET1703-96	huabio	1:1000 (WB)
GLUL	66323-1-Ig	proteintech	1:1000 (WB)
CDK4	11026-1-AP	proteintech	1:1000 (WB)
IgG	A7050-2mg	beyotime	

Supplement table 4. Sequence information of primers used in the study

RT-qPCR primers	Sequence (5′–3′)
GAPDH-F	CATCACCATCTTCCAGGAGCGAGA
GAPDH-R	TGCAGGAGGCATTGCTGATGATCT
TMEM209-F	CCCTTGCATCTCTCTTCAGCC
TMEM209-R	CCAGGACTAACAACCAGACTTG

KPNB1-F	TCACTAGGGATCTGCACCCA
KPNB1-R	CCCAACAGGAGATGGACACC
