# Supplementary material for: TMEM209 promotes hepatocellular carcinoma progression by activating the Wnt/β-catenin signaling pathway through KPNB1 stabilization
Source: Cell Death Discov. 2024 Oct 16;10:438. doi: 10.1038/s41420-024-02207-9 (PMC11484822; doi:10.1038/s41420-024-02207-9)

Original western-blot bands

Figure 1D

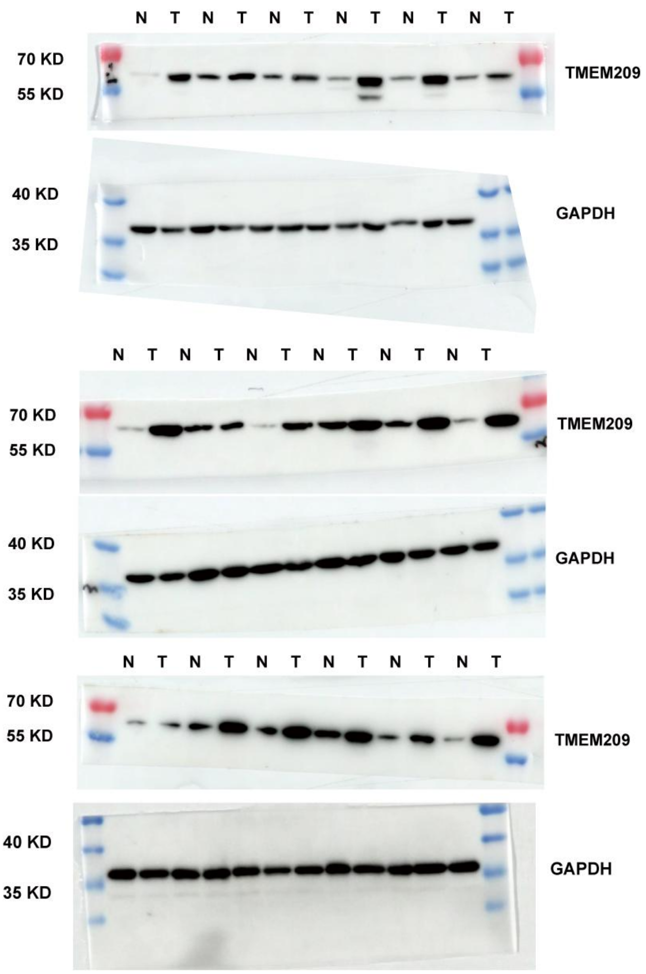

Figure 2A

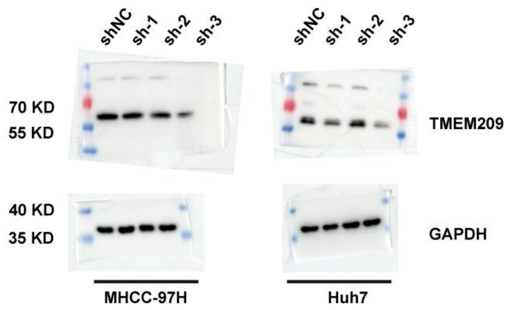

Figure 2G

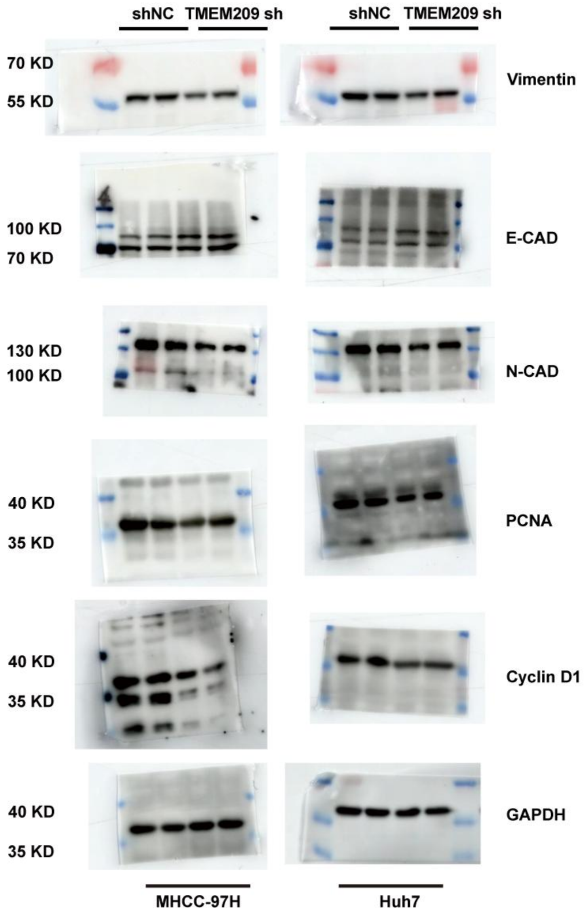

Figure 3C

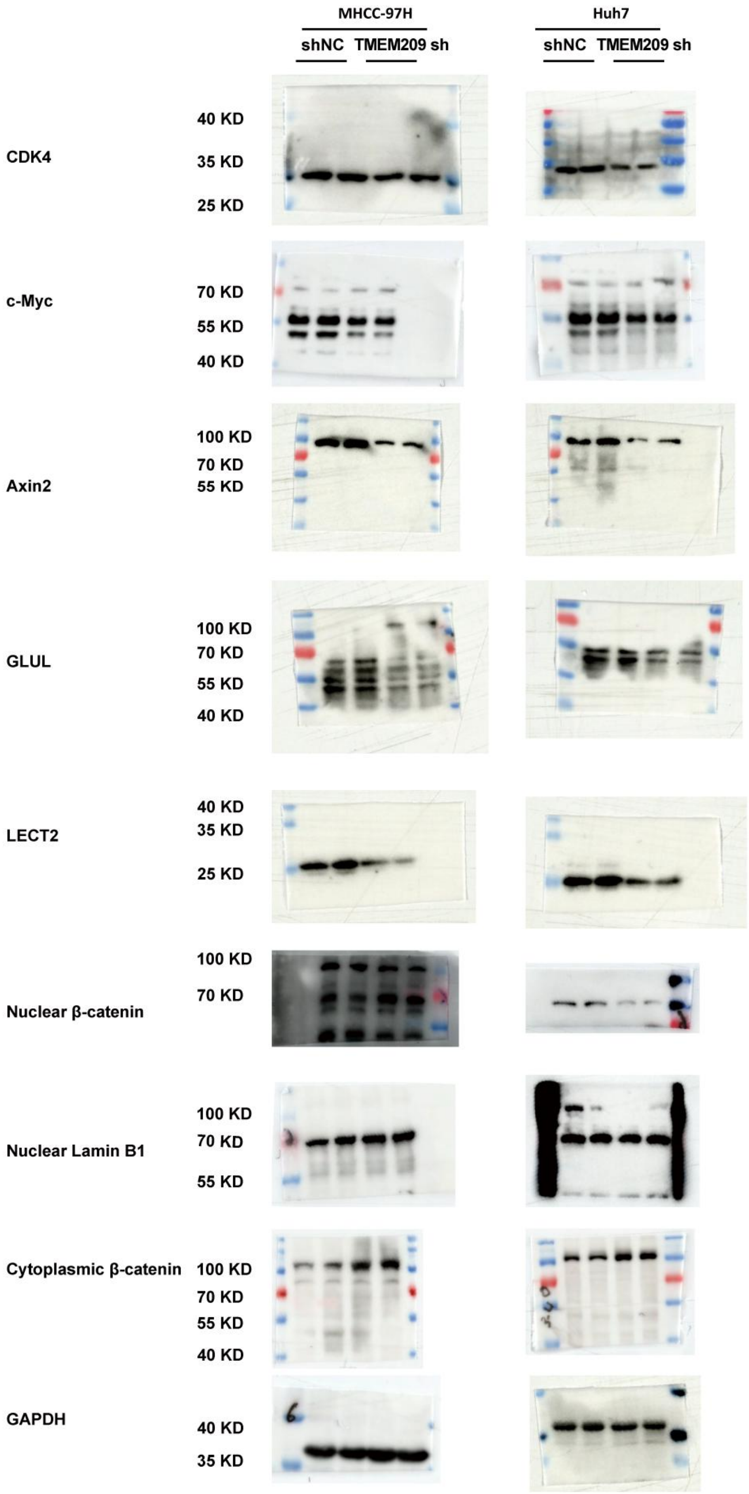

Figure3D

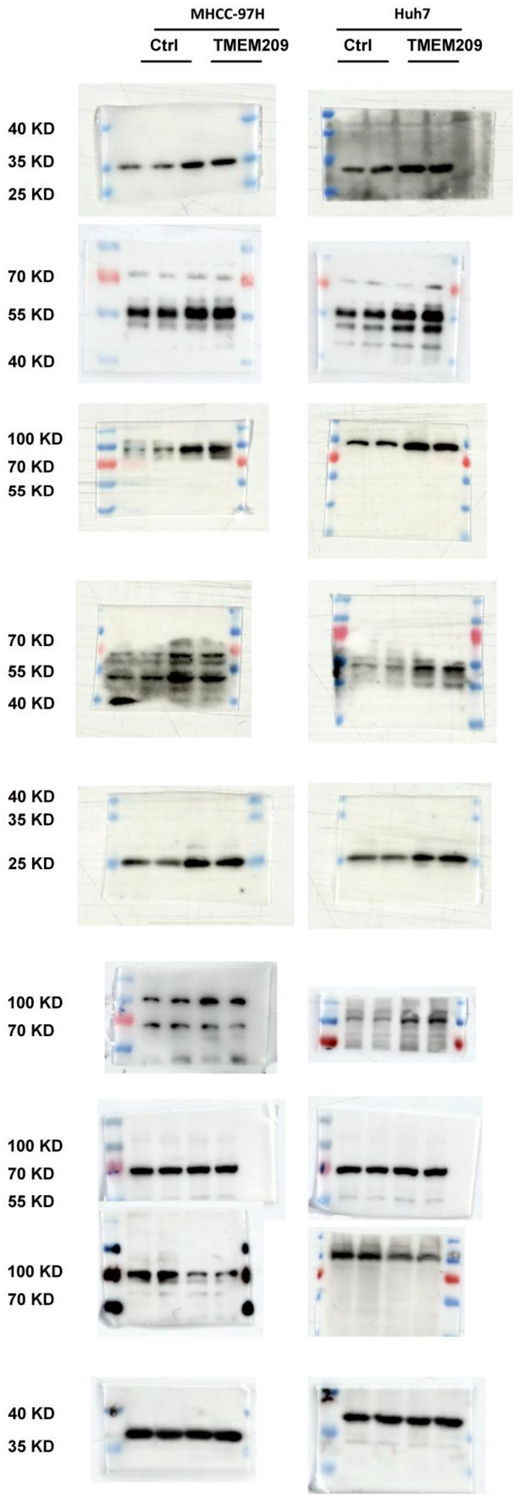

Figure 4B

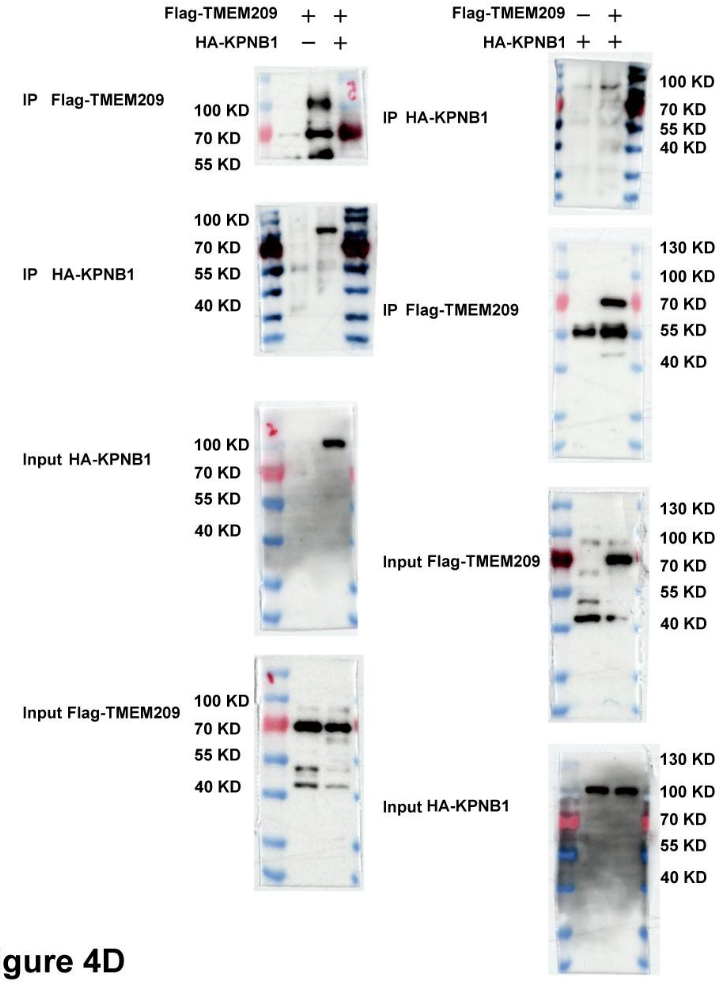

Figure 4C

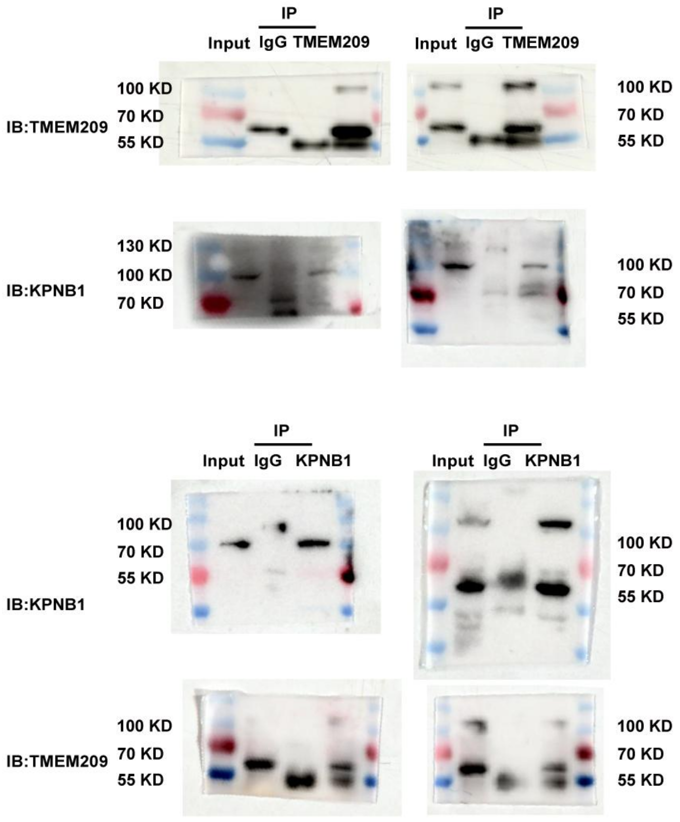

Figure 4D

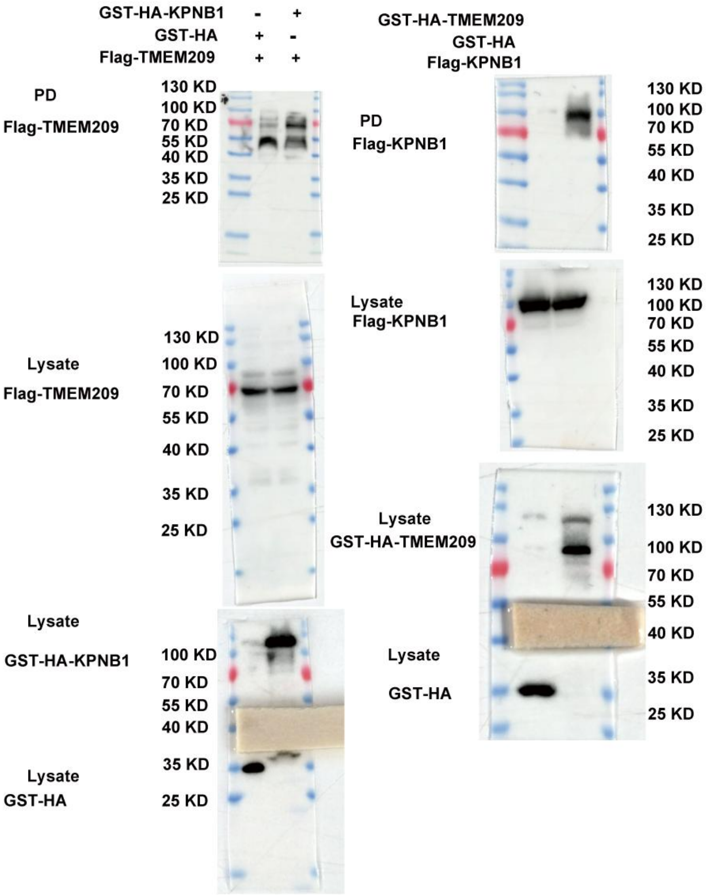

Figure 4E

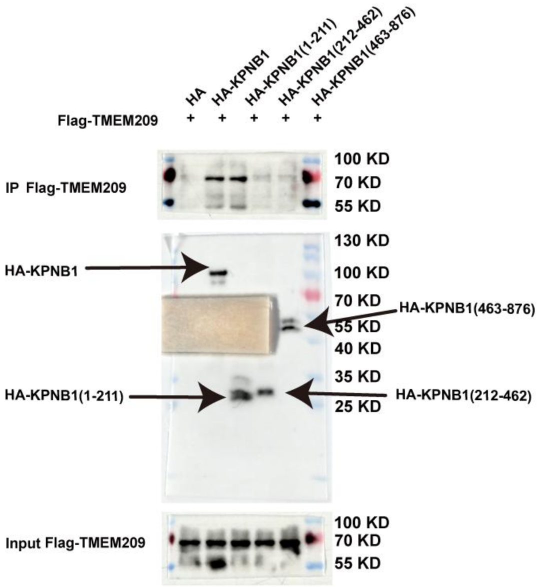

Figure 4G

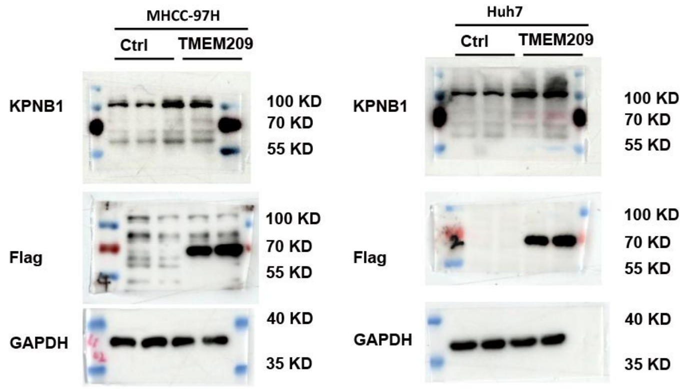

Figure 5A

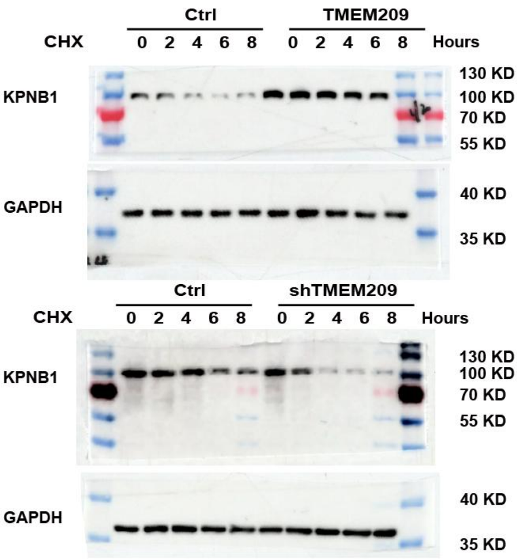

Figure 5B

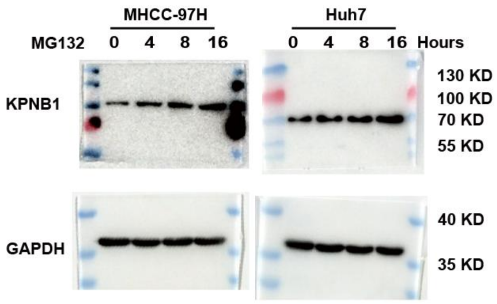

Figure 5C

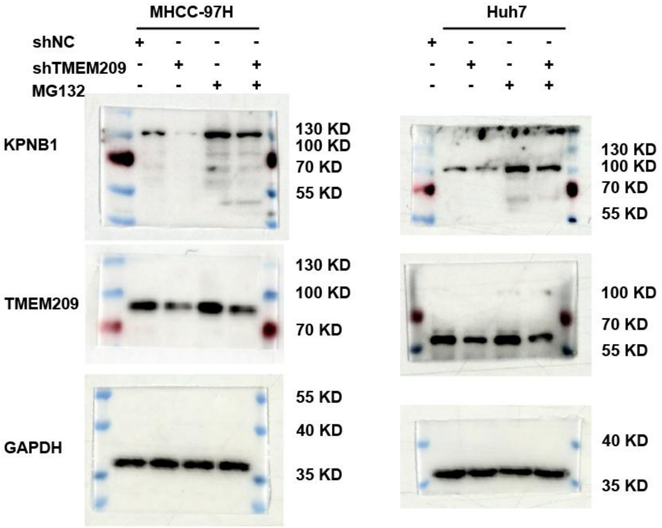

Figure 5D

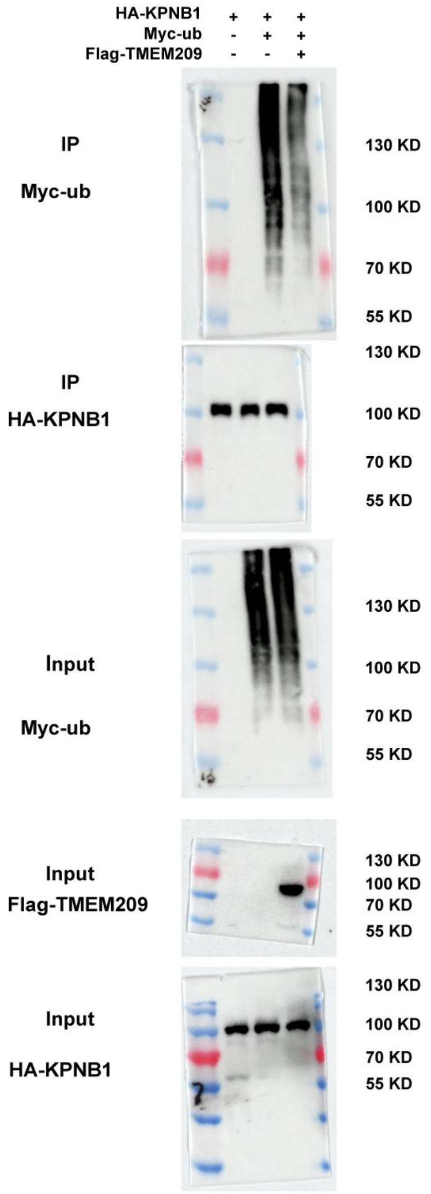

Figure 5E

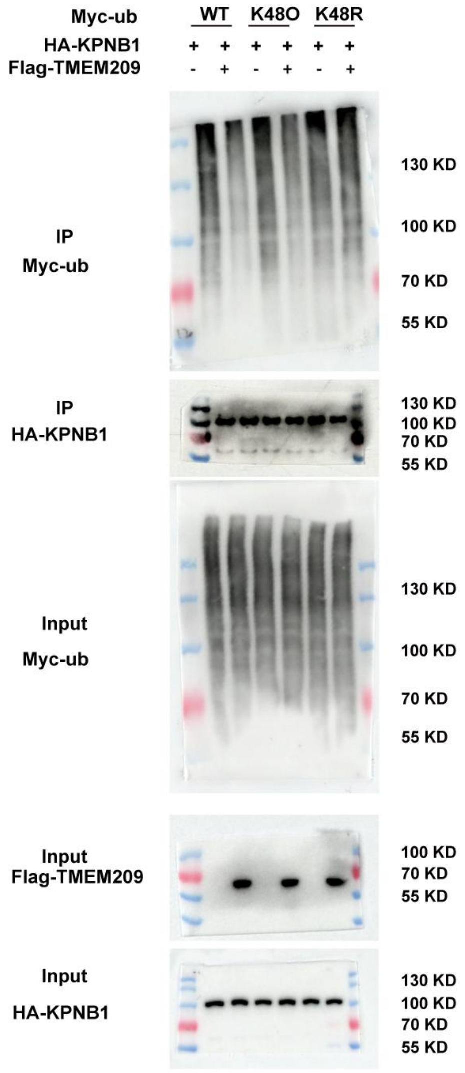

Figure 5G

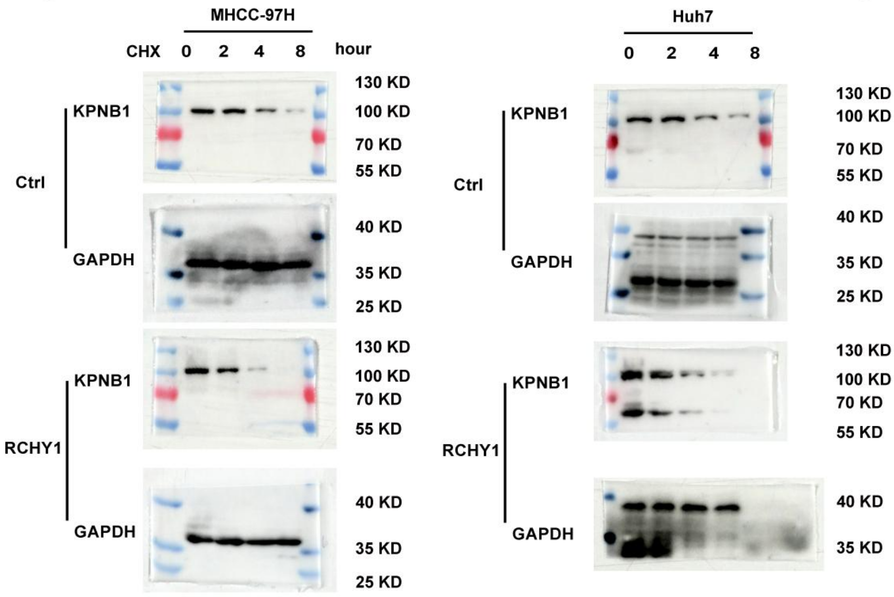

Figure 5H

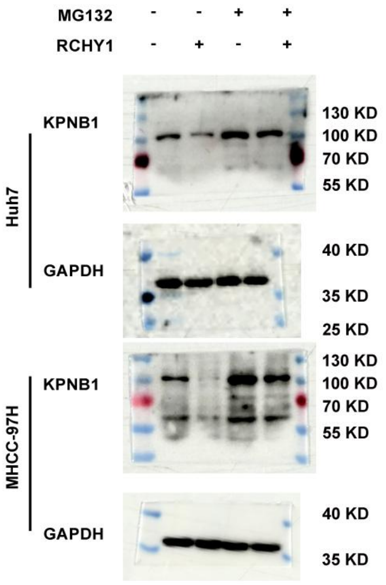

Figure 5I

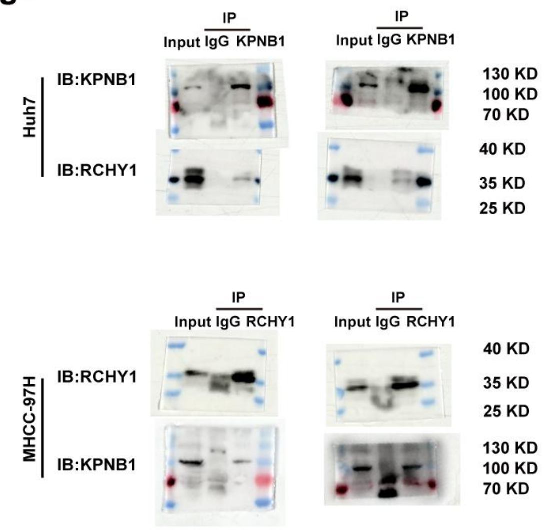

Figure 5J

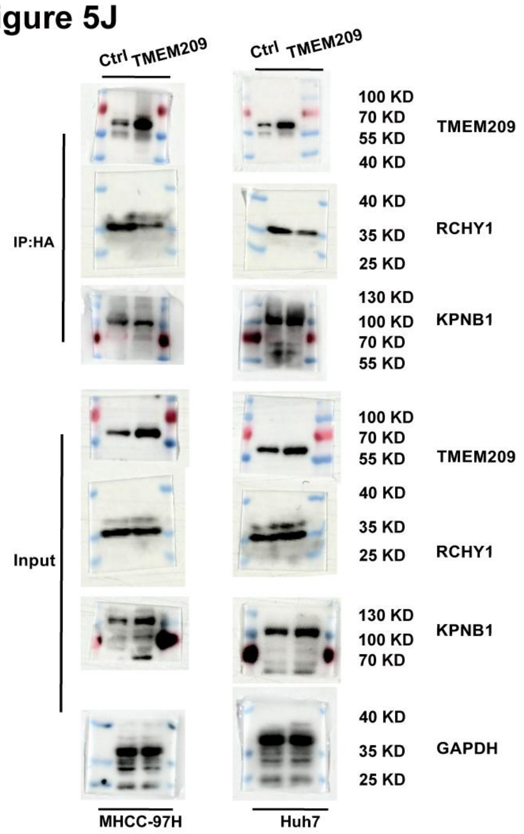

Figure 5K

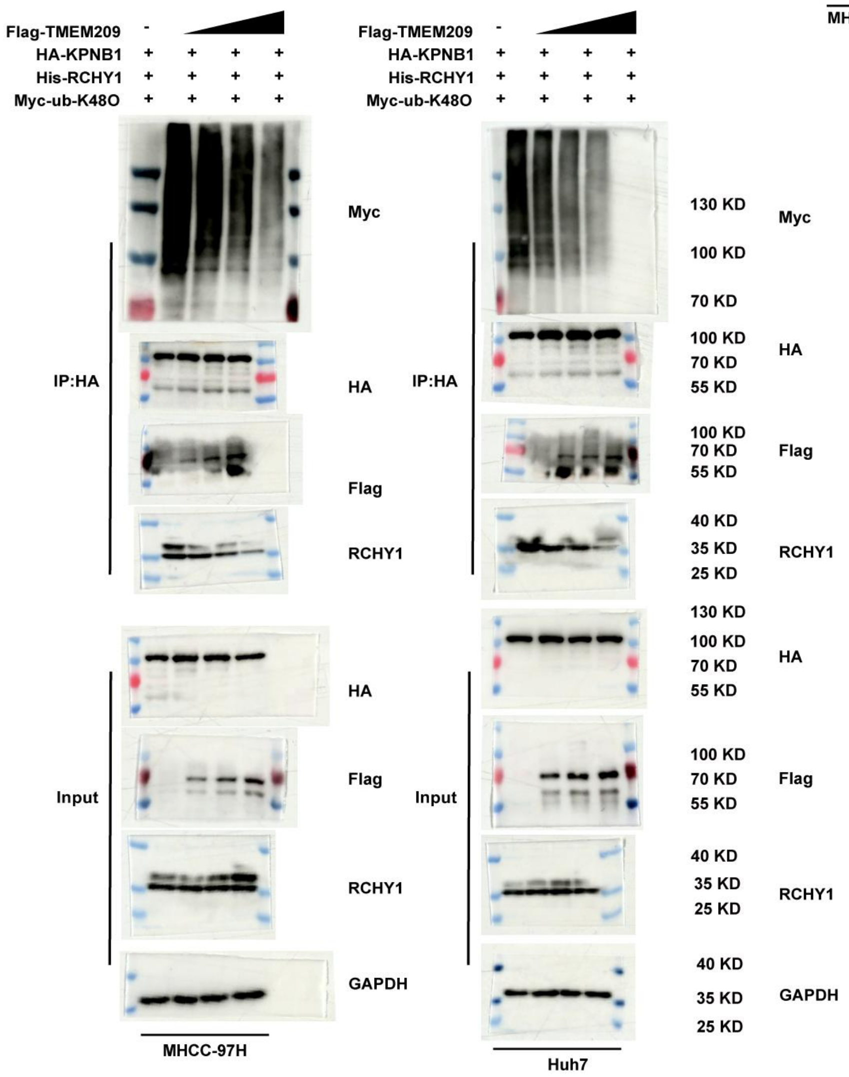

Figure 6A

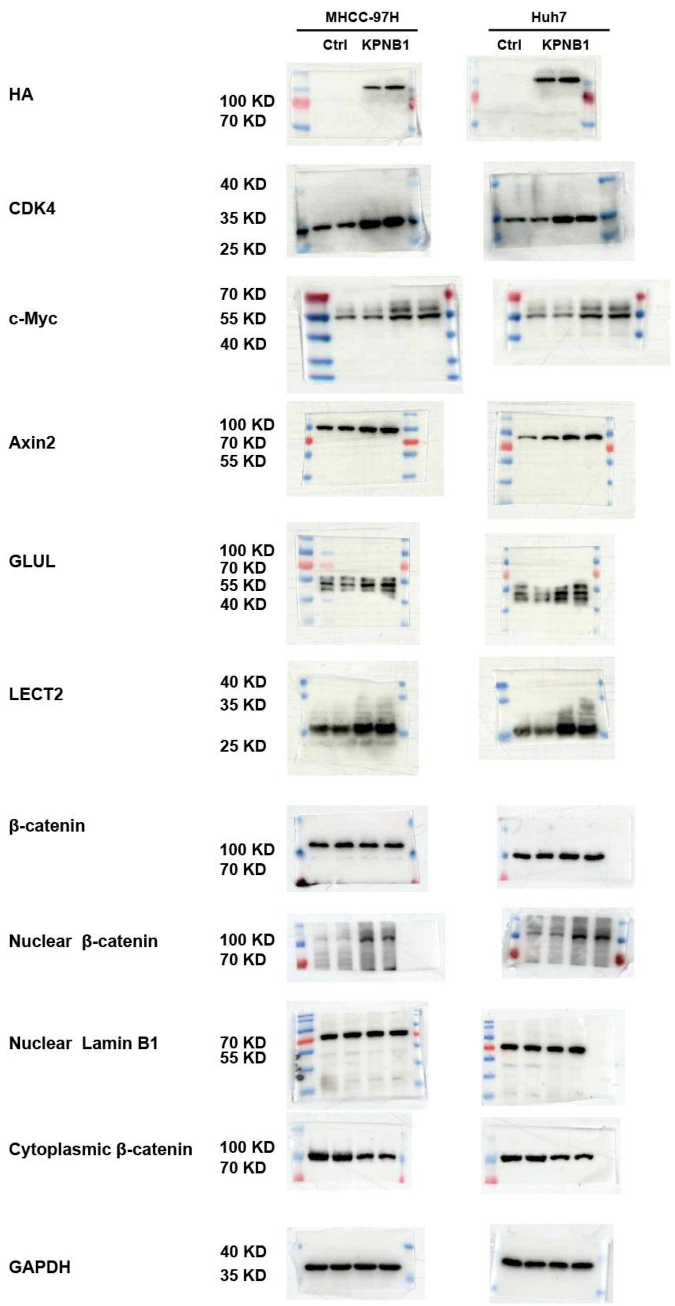

Figure 6B

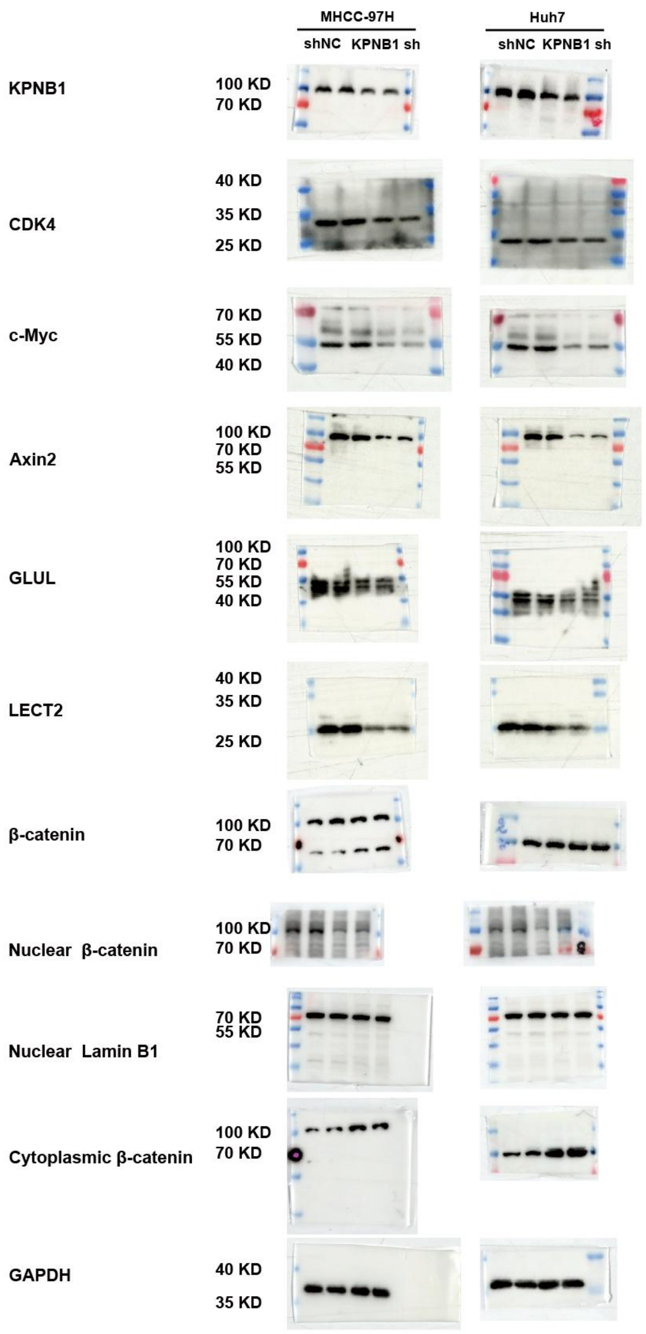

Figure 7A

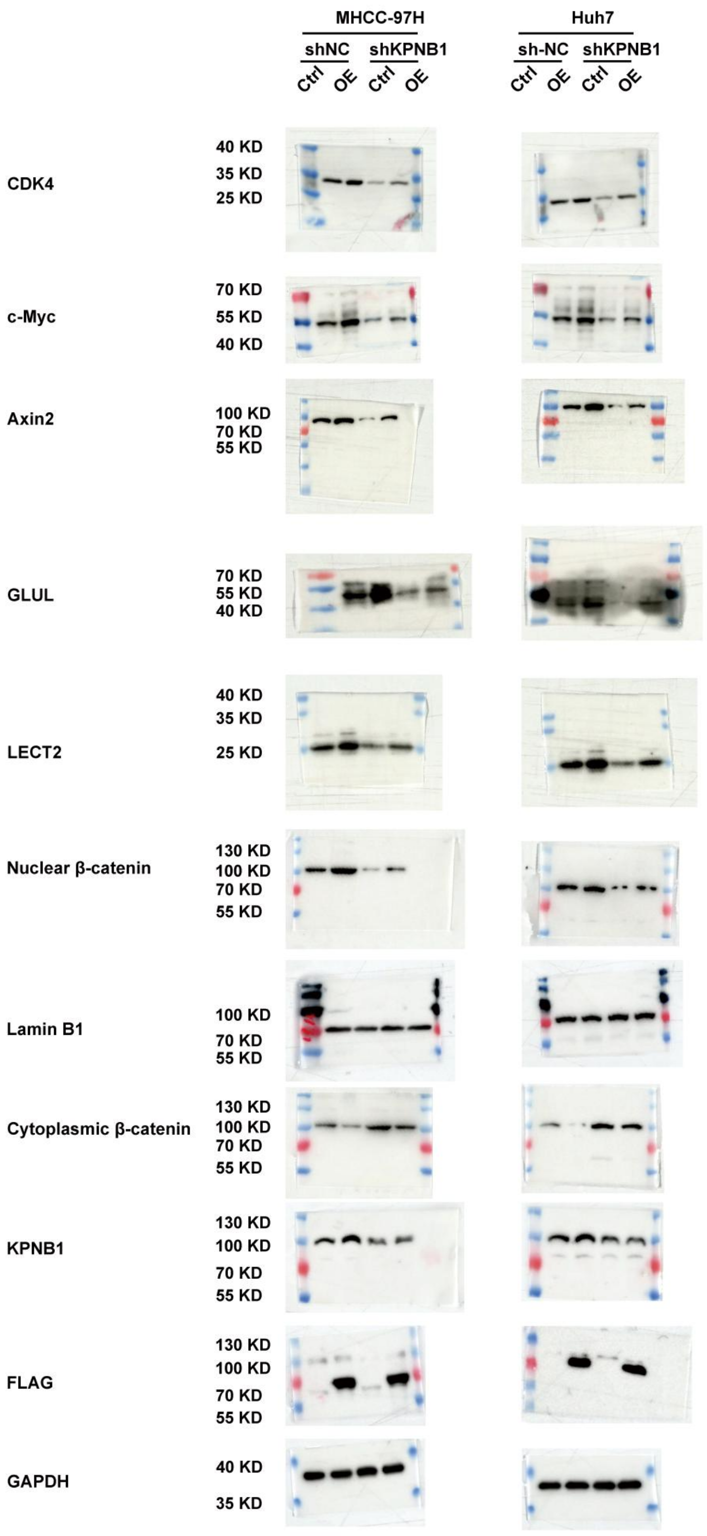

MHCC-97H
Huh7
shNC
shKPNB1
sh-NC
shKPNB1
Ctrl
OE
Ctrl
OE
CDK4
40 KD
35 KD
25 KD
c-Myc
70 KD
55 KD
40 KD
Axin2
100 KD
70 KD
55 KD
GLUL
70 KD
55 KD
40 KD
LECT2
40 KD
35 KD
25 KD
Nuclear β-catenin
130 KD
100 KD
70 KD
55 KD
Lamin B1
100 KD
70 KD
55 KD
Cytoplasmic β-catenin
130 KD
100 KD
70 KD
55 KD
KPNB1
130 KD
100 KD
70 KD
55 KD
FLAG
130 KD
100 KD
70 KD
55 KD
GAPDH
40 KD
35 KD

Supplment Figure 2A

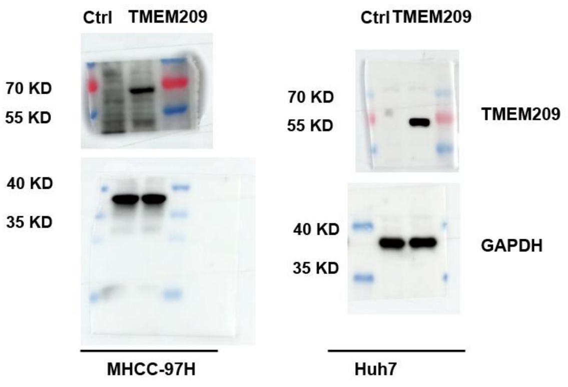

Supplment Figure 2G

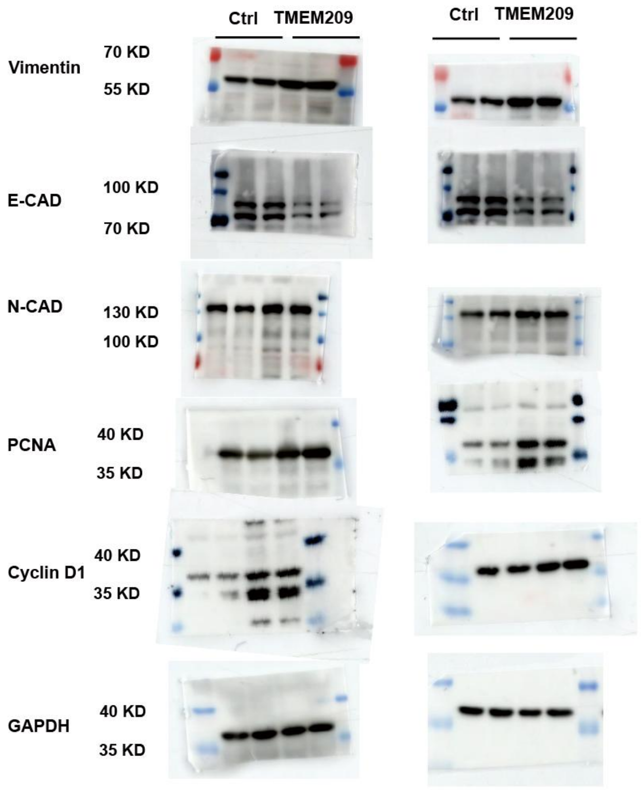

Supplment Figure 3A

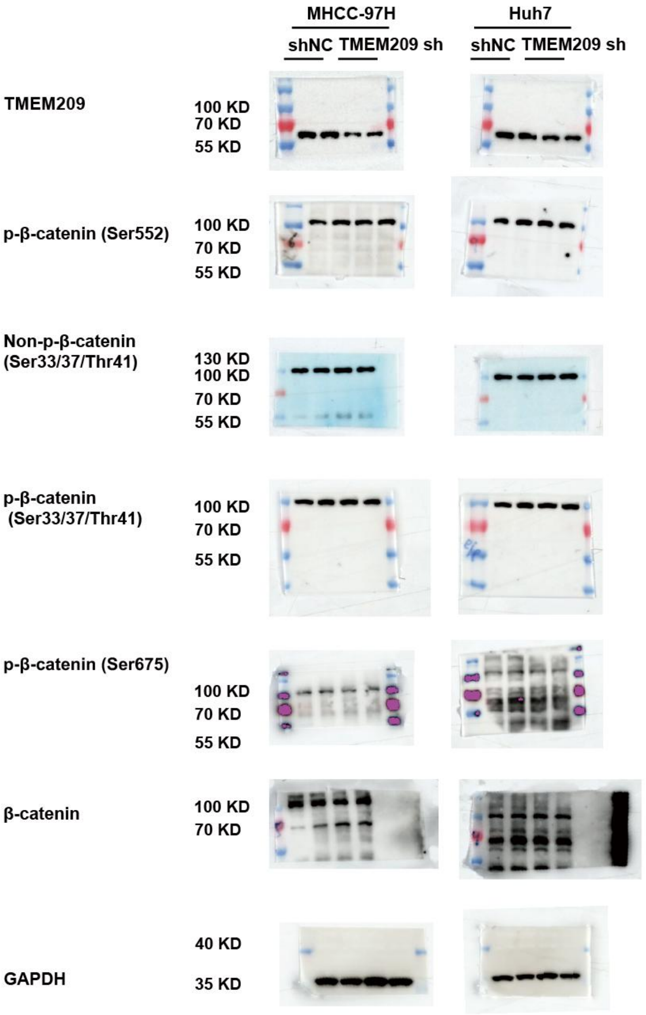

Supplment Figure 3B

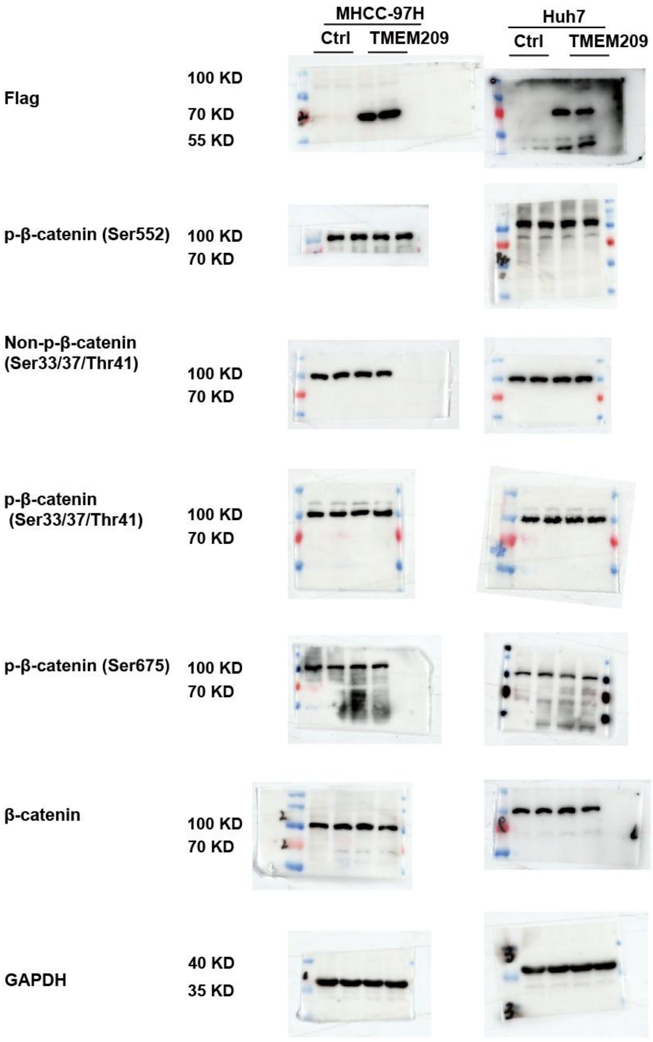

Supplement Material 7B

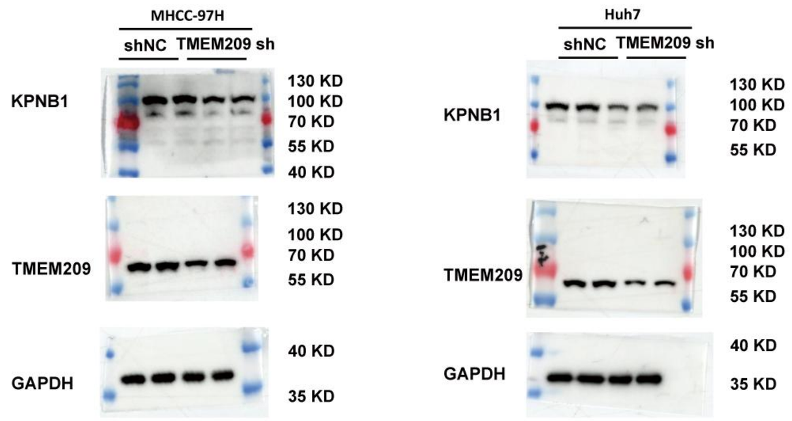

Supplement Material 8A

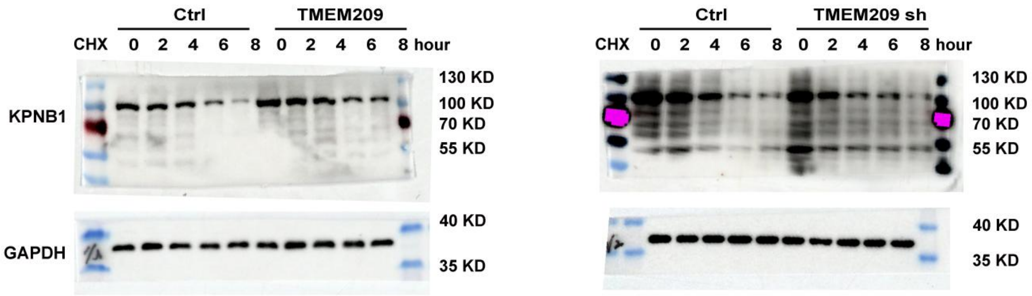

Supplement Material 8B

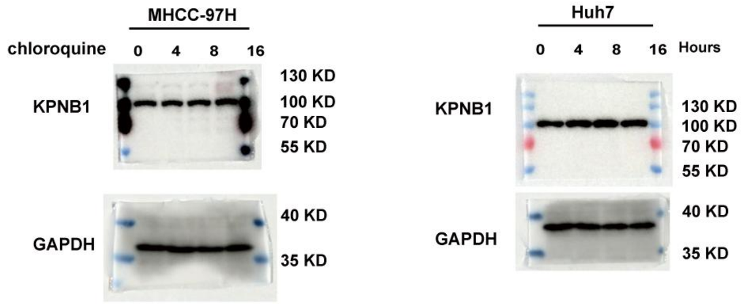

Supplement Material 9A

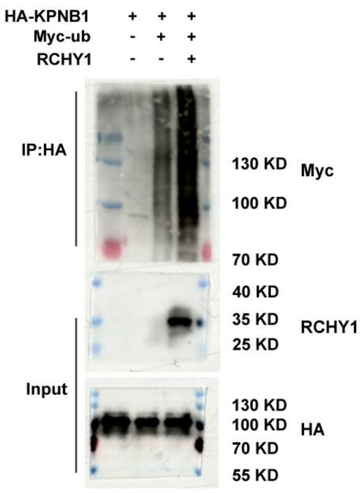

Supplement Material 9B

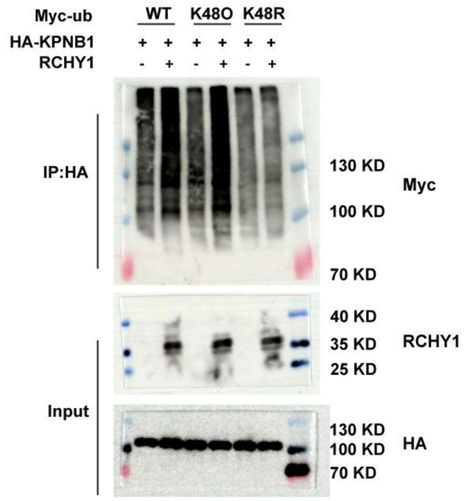

Supplement: Supplementary file 2 — Original western blot brands [file 41420_2024_2207_MOESM2_ESM.pdf]
